# N-Acetylcysteine in the Treatment of Acute Lung Injury: Perspectives and Limitations

**DOI:** 10.3390/ijms26062657

**Published:** 2025-03-15

**Authors:** Daniela Mokra, Igor Porvaznik, Juraj Mokry

**Affiliations:** 1 Department of Physiology, Jessenius Faculty of Medicine in Martin, Comenius University in Bratislava, SK-03601 Martin, Slovakia; 2 Department of Laboratory Medicine, Faculty of Health Sciences, Catholic University in Ružomberok, SK-03401 Ružomberok, Slovakia; igor.porvaznik@ku.sk; 3 Department of Pharmacology, Jessenius Faculty of Medicine in Martin, Comenius University in Bratislava, SK-03601 Martin, Slovakia; juraj.mokry@uniba.sk

**Keywords:** acute lung injury, ARDS, COVID-19, N-acetylcysteine

## Abstract

N-acetylcysteine (NAC) can take part in the treatment of chronic respiratory diseases because of the potent mucolytic, antioxidant, and anti-inflammatory effects of NAC. However, less is known about its use in the treatment of acute lung injury. Nowadays, an increasing number of studies indicates that early administration of NAC may reduce markers of oxidative stress and alleviate inflammation in animal models of acute lung injury (ALI) and in patients suffering from distinct forms of acute respiratory distress syndrome (ARDS) or pulmonary infections including community-acquired pneumonia or Coronavirus Disease (COVID)-19. Besides low costs, easy accessibility, low toxicity, and rare side effects, NAC can also be combined with other drugs. This article provides a review of knowledge on the mechanisms of inflammation and oxidative stress in various forms of ALI/ARDS and critically discusses experience with the use of NAC in these disorders. For preparing the review, articles published in the English language from the PubMed database were used.

## 1. Introduction

NAC is a widely used therapeutic agent because of its potent antioxidant and mucolytic action. In addition, NAC is useful as an antidote for acetaminophen (paracetamol) intoxication. However, NAC also possesses numerous anti-inflammatory, anti-fibrotic, and cytoprotective effects which favor its use in the treatment of various inflammatory disorders including pulmonary diseases.

There is an increasing body of evidence indicating that early administration of NAC may reduce oxidative stress and mitigate inflammation in both animal models of ALI and in patients suffering from distinct forms of ARDS including community-acquired pneumonia or COVID-19. The advantages of using NAC are mainly its low cost, wide availability, low toxicity, and rare side effects. Moreover, animal studies showed that NAC can also be easily combined with other drugs with anti-inflammatory effects such as corticosteroids and statins, or with exogenous surfactant.

This article provides a review of knowledge on the mechanisms of inflammation and oxidative stress in various forms of ALI/ARDS and critically discusses experience with the use of NAC in these disorders. For preparing the review, articles published in English from the PubMed database were used.

## 2. ALI and ARDS

### 2.1. Definitions of ALI and ARDS

Acute respiratory distress with the typical clinical picture of tachypnea, severe cyanosis unresponsive to oxygen administration, decreased lung compliance, and the presence of diffuse alveolar infiltrates on chest X-ray was first described in 1967 by Ashbaugh et al. [1]. Later, the American–European Consensus Conference in 1994 defined the following criteria: (a) acute onset of symptoms after a known risk factor with a maximum within a week; (b) severe hypoxemia resistant to oxygen therapy, with a more severe form of respiratory insufficiency defined by a ratio between arterial partial pressure of oxygen and fraction of inspired oxygen (PaO_2_/FiO_2_) ≤ 200 mmHg (26.7 kPa) named “Acute Respiratory Distress Syndrome (ARDS)”, and a milder form of this syndrome with PaO_2_/FiO_2_ 200–300 mmHg (40 kPa) named “acute lung injury (ALI)”; (c) diffuse bilateral infiltrates on chest X-ray; (d) absence of cardiogenic pulmonary edema verified by wedge pressure in the pulmonary artery ≤ 18 mmHg or absence of clinical symptoms of hypertension of the left ventricle [2]. More recently, the so-called Berlin Classification in 2012 defined three degrees of ARDS according to hypoxemia at a level of positive end-expiratory pressure (PEEP) of ≥5 cmH_2_O (0.5 kPa): (a) mild ARDS with PaO_2_/FiO_2_ between 200 and 300 mmHg, (b) moderate ARDS with PaO_2_/FiO_2_ between 100 and 200 mmHg, and (c) severe ARDS with PaO_2_/FiO_2_ ≤ 100 mmHg. The term “acute lung injury” was omitted from the Berlin definition and nowadays it has been used as a general term describing acute lung damage or for experimental models where the clinical criteria of the definition cannot be fulfilled [2,3,4].

### 2.2. Epidemiology and Mortality of ARDS

The incidence of ARDS varies due to regional genetic variability, differences in health care systems, and different diagnostic criteria for ARDS [5]. The multicenter prospective study carried out in 459 intensive care units in 50 countries in 5 continents revealed that ARDS represented 10.4% of total admissions to ICU and 23.4% of all patients requiring mechanical ventilation. The prevalence was 30.0% for mild ARDS, 46.6% for moderate ARDS, and 23.4% for severe ARDS, and overall, unadjusted ICU and hospital mortality was 35–45%, in relation to ARDS severity [6]. These findings agree with the Berlin definition, in which the estimation of mortality based on increasing severity resulted in 27% mortality in mild ARDS, 32% mortality in moderate ARDS, and 45% mortality in severe ARDS [3]. The ARDS mortality may be attributable to the respiratory failure itself, or to sepsis and multiple organ failure [7]. The recent COVID-19 pandemic has substantially increased the prevalence of ARDS and highlighted challenges linked with this syndrome, including its high mortality and lack of effective pharmacotherapy [8].

Most ARDS survivors may recover to normal or near-normal lung functions. However, many of them remain burdened by functional limitations because of marked and persistent muscle weakness, polyneuropathy, sensorineural hearing loss, etc., or because of mental disorders such as depression, anxiety, post-traumatic stress disorder, or cognitive impairment [9,10].

### 2.3. Causes and Risk Factors of ARDS

ARDS may develop due to direct (pulmonary) or indirect (extrapulmonary) insults. The most common causes of direct ARDS are pneumonia (mainly of bacterial or viral origin), aspiration of gastric contents, near-drowning, inhalation of toxic gases or smoke, etc. The indirect ARDS may origin due to non-pulmonary sepsis, severe trauma with shock, pancreatitis drug abuse, burns, etc. [11]. However, other factors increasing the risk of ARDS have been recognized such as genetic factors determining susceptibility or resistance to ARDS, virulence of the pathogen, environmental factors (cigarette smoke exposure and environmental pollution), older age (>65 years), race, sex, chronic alcohol abuse, concomitant chronic diseases, etc., [12,13].

### 2.4. Major Pathomechanisms of ARDS

The underlying pathomechanisms of ARDS have been previously discussed in numerous excellent reviews [8,11,12,14]. Therefore, this article brings only the basal information that is necessary to understand the issues of inflammation-related oxidative stress in ARDS as a rationale for the use of antioxidants, including NAC, in the treatment of this syndrome.

ARDS usually develops in two phases: the acute (or exudative) phase which occurs within the first 7 days after initiating insult and the resolution (or proliferative) phase which follows the initial phase [14]. In half of the patients, the exudative phase may be linked with diffuse alveolar damage leading to severe interstitial and alveolar edema, associated with higher mortality [15]. Acute alveolar injury results from the alteration of both epithelial and endothelial cells. Epithelial cell destruction, cell necrosis/apoptosis, and denudation of the alveolar basement membrane are responsible for edema and alveolar hemorrhage. In addition, injured epithelial cells may enhance the production of procoagulant factors and the deposition of intra-alveolar fibrin [14].

The alteration of endothelial cells includes a functional breakdown in endothelial junctions and activation of the cells by inflammatory signals from microbes (toxins), damaged cells (endogenous danger-associated molecular patterns), or activated immune cells (e.g., tumor necrosis factor (TNF)α, interleukin (IL)-1β as a product of the inflammasome activation, angiopoietin 2, vascular endothelial growth factor (VEGF), platelet-activating factor (PAF), etc.) [12]. Activated endothelial cells generate various bioactive substances (e.g., angiopoietin 2) and stimulate the accumulation of leukocytes, eventually forming neutrophil-platelet aggregates, in the lungs, increasing vascular permeability for proteins [12].

An additional burden is linked with the excessive recruitment and activation of immune cells, particularly neutrophils. These cells release huge amounts of proteolytic enzymes (such as neutrophil elastase) and reactive oxygen species (ROS) and form neutrophil extracellular traps (NETs), which contribute to endothelial and epithelial damage [12]. The activation of neutrophils is often associated with platelets, important not only in triggering NETs but also in thrombogenic activity. In addition to neutrophils, intra-alveolar macrophages generate potent chemotactic factors such as IL-8 and monocyte chemoattractant protein (MCP)-1 attracting additional neutrophils and monocytes from the blood into the lungs. Despite being more resistant to injury than the endothelium, the extent of epithelial injury in the early phase of ARDS is an important determinant of the severity of ARDS [14].

Clinical recovery is strongly dependent on the repair of injured epithelium during the proliferative phase of ARDS which may last several days up to several weeks. Epithelial repair is based on the proliferation of alveolar cells type II and their trans-differentiation to type I cells under the complex influence of growth factors and co-operating cells (fibroblasts, endothelial cells, and epithelial cells) [16]. Once the alveolar epithelium is restored, pro-resolving macrophages remove the dead cells and debris and alveolar epithelial cells reabsorb the edematous fluid. Unfortunately, in some cases, the repair process does not lead to complete healing as dysregulated fibroblasts produce excessive amounts of growth factors and deposit collagen, leading to interstitial fibrosis [14].

Summarizing, the severity of epithelial and endothelial injury is a major determinant of clinical outcome in ARDS. Some authors speculate that the direct (i.e., pulmonary) and indirect (i.e., non-pulmonary) causes of ARDS may be pathologically distinct. Supporting this hypothesis, higher plasma levels of biomarkers of lung epithelial injury such as receptor for advanced glycation end products (RAGE) and surfactant protein D were found in the patients with direct lung injury, whereas higher plasma biomarkers of endothelial injury and inflammation were detected in the patients with indirect lung injury (including angiopoietin 2) [17]. An alternative approach has differentiated a hyperinflammatory subphenotype in about 30% of the patients which was typical with high plasma levels of inflammatory biomarkers (IL-6, IL-8, and soluble TNF receptor 1), low protein C, high prevalence of shock and metabolic acidosis, and higher mortality, and a hypoinflammatory subphenotype in about 70% of the patients which was characterized by lower levels of the inflammatory biomarkers and lower occurrence of acidosis and vasopressor-dependent shock [18,19,20]. In addition, these subphenotypes responded differently to PEEP, fluid management, and simvastatin therapy [20]. The mentioned data suggest that further research is needed to identify specific patterns that may help to segregate pathways of lung and systemic injury or direct and indirect ARDS, respectively [14].

### 2.5. ARDS Due to Severe Viral Diseases and COVID-19

As previously mentioned, oxidative stress is strongly involved in the pathogenesis of ARDS. One of the direct pulmonary ARDS is pneumonia resulting from respiratory viral disease, e.g., Severe Acute Respiratory Syndrome Coronavirus (SARS-CoV-)2 infection. In general, respiratory viral infections are linked with the activation of pro-inflammatory and pro-oxidative pathways including nuclear factor (NF)-κB signaling, and the inhibition of anti-inflammatory and anti-oxidative pathways including nuclear factor erythroid-derived 2-like (Nrf)2 signaling may lead to uncontrolled inflammation and redox imbalance [21]. The overproduction of ROS and deprivation of antioxidant mechanisms contribute not only to viral replication [22], but are also implicated in inflammation, lung epithelial disruption, tissue damage, and cell death resulting in macrophage activation. Moreover, oxidative stress triggers an antiviral immune response, which in case of excessive activation may lead to a “cytokine storm” and severe inflammation [21]. As the depletion of glutathione (GSH) has been recognized as a key mechanism of redox imbalance in viral infections that favors viral replication, the substitution of GSH via the administration of NAC seems to be a reasonable approach [23].

#### Pathophysiology of COVID-19

Statistics have shown that infection with the SARS-CoV2 virus caused no symptoms or mild COVID-19 disease in most patients, while 14% of the patients suffered from a severe course of the disease, and a critical condition developed in 5% of the patients, which in up to 50% of them ended fatally [24]. The majority of patients with severe COVID-19 disease had some associated comorbidities, such as diabetes mellitus, obesity, or cardiovascular and immunosuppressive diseases. These are linked with chronic inflammation and higher basal levels of ROS leading to dysfunction of endothelial and epithelial glycocalyx, which contributed to a worse course and increased mortality from COVID-19 in these patients [21,25].

While the pathophysiology of COVID-19 has some peculiarities, those specifics that are essential for understanding the rest of the text are explained in this section. After the SARS-CoV-2 virus enters the body, mechanisms of innate non-specific immunity, such as physiological barriers, neutrophils, monocytes/macrophages, and dendritic and mast cells, are involved in the initial response. Later, the mechanisms of specific immunity become also engaged, with the activation of B- and T-lymphocytes, formation of antibodies, and production of cells that are able to destroy the virus-infected cells [26].

For entry into the cell, SARS-CoV-2 binds to the host receptors, i.e., angiotensin-converting enzyme (ACE)2 and transmembrane protease serine (TMPRSS)2. Because of the highest ACE2 receptor distribution, the lungs are primarily affected, followed by the digestive tract, heart, kidneys, and brain [27]. After entering the cell, the virus releases its RNA in the cytoplasm, which is recognized by receptors for pathogen-associated molecular patterns (PAMPs). These receptors activate NF-κB which subsequently translocates to the nucleus, where it induces the transcription of pro-inflammatory cytokines and chemokines including interferon (IFN) I [26]. Interferons play a key role in the initiation of the inflammatory response. During active viral replication, elevated levels of IFN I inhibit the viral replication and act as a chemoattractant for immune cells. SARS-CoV-2 causes a slowdown in IFN I secretion, especially in elderly patients and patients with comorbidities, which reduces chemotaxis and leads to a weaker immune response [28]. It also triggers faster viral replication and overactivation of Th1 cells with higher IFN II (or IFNγ) production. As a result, activated macrophages generate high amounts of IL-1, IL-6, IL-8, and transforming growth factor (TGF)-β, also stimulating Th17 cells producing IL-17.

Together, these changes lead to the so-called “cytokine storm”, which is associated with enormous oxidative stress and the depletion of the antioxidant system [29]. High levels of oxidants damage the cell membrane and thereby reduce the functionality of CD4+ T-lymphocytes and NK-cells, which also suppresses the antiviral activity of CD8+ T-lymphocytes and lowers the neutralizing capacity of antibodies [30]. Moreover, excessive oxidative stress induces the activation of NF-κB in T-lymphocytes and macrophages, which stimulates the production of pro-inflammatory cytokines, especially TNFα and IL-6 [26]. The activation of NF-κB, elevation of angiotensin II, and oxidative stress in COVID-19 also stimulate the intracellular NLR family pyrin domain containing 3 (NLRP3) inflammasome. This cytosolic multiprotein complex is able to turn on the inflammatory response via activation of caspase-1 and conversion of IL-1β and IL-18 from their precursors and to induce pyroptosis, a special type of cell death associated with intense inflammation [31,32].

Although the clinical picture of COVID-19 is mainly represented by the pulmonary symptoms, the vascular damage and increased formation of thrombi are crucial factors in the development of a severe disease. Endothelial dysfunction in COVID-19 is attributable to multiple factors [33]. SARS-CoV-2 prevents the conversion of angiotensin II to angiotensin 1–7, which may counterbalance the detrimental effects of angiotensin II, generated by ACE2. This leads to increased production of superoxide, nitric oxide (NO), and peroxynitrite in various cells via the activation of nicotinamide adenine dinucleotide phosphate (NADPH) oxidase and NO synthase [34]. All together, the previously mentioned factors result in endothelial dysfunction and local tissue damage. Furthermore, the SARS-CoV-2 virus spike protein itself directly induces mitochondrial fusion and reduces endothelial NO synthase (eNOS) activity, thereby contributing to damage of endothelial cells in various organs [35]. Endothelial injury, platelet activation, and release of coagulation factors leading to a pro-thrombotic state enhance the release of pro-inflammatory cytokines, generating a vicious cycle [36].

The above-mentioned inflammatory and oxidative changes including inflammasome activation result in multiorgan damage with not only respiratory but also cardiovascular and neurological manifestations which may persist for a long time [37,38]. The neurological symptoms of COVID-19 likely originate from SARS-CoV-2 infection of the olfactory epithelium expressing ACE2 receptor that leads to anosmia, as well as from the virus-induced neuroinflammation and oxidative stress damaging biomolecules in the cells including neurons that cause headaches, depression, cognitive impairments, or “brain fog” [39].

The elucidation of the prominent role of oxidative stress in the pathogenesis of COVID-19 has suggested the perspective of implementing various antioxidative strategies including NAC in the prevention and treatment of COVID-19.

### 2.6. Standard Treatment of ARDS

Lung-protective ventilation with PEEP matching the fraction of inspired oxygen [40,41] remains a fundamental approach in the management of ARDS. Although it does not cure ARDS, its use provides a time window for recovery from respiratory failure and supplies appropriate oxygenation and carbon dioxide removal [8]. Prone positioning is another approach potentially improving oxygenation thanks to the mitigation of regional heterogeneity in the distribution of ventilation [42,43]. In an attempt to control oxygen consumption, additional approaches such as reducing body temperature [44], sedation [45], and neuromuscular blockade [46] may be used. Standard therapy of ARDS also includes supportive care in terms of fluid-conservative management [47], caloric supplementation, and management of pain [8].

On the other hand, numerous trials testing the efficacy of various pharmacotherapies have failed [48,49,50]. These inconsistent results are likely a consequence of the significant heterogeneity of ARDS, clearly evident in its various causes, manifestations, and response to therapy [4,18,20], challenging clinicians and researchers to discover new therapies that may be potentially beneficial [8]. For instance, the use of antioxidants is supported by the finding of inflammation-related oxidative stress in ARDS. However, the data from large trials were rather inconsistent and did not demonstrate any positive response to the administration of vitamin D [51,52] or vitamin C [53,54]. Thus, the goal of this article was to review the efficacy of another antioxidant, NAC, in various animal models of ALI as well as in clinical ARDS including COVID-19.

## 3. Pharmacological Effects of NAC Potentially Beneficial in ARDS

**Figure 1:**
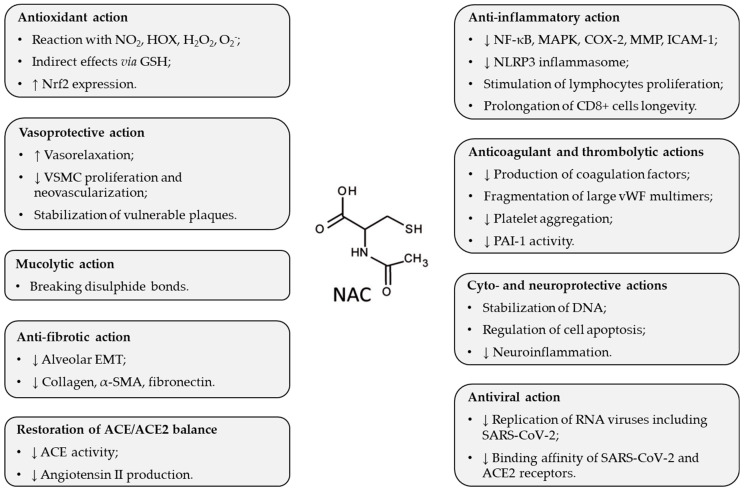
Major actions of NAC useful in ARDS. Abbreviations: ACE: angiotensin-converting enzyme; COX-2: cyclooxygenase; DNA: deoxyribonucleic acid; EMT: epithelial–mesenchymal transition; GSH: glutathione; H_2_O_2_: hydrogen peroxide; HOX: hypohalous acids; ICAM-1: intercellular adhesion molecule; MAPK: mitogen-activated protein kinases; MMP: matrix metalloproteinases; NF-κB: nuclear factor kappa B; NLRP3: NLR family pyrin domain containing 3; NO_2_: nitrogen dioxide; Nrf2: nuclear factor erythroid-derived 2-like 2; O_2_^−^: superoxide anion; PAI-1: plasminogen activator inhibitor; RNA: ribonucleic acid; SARS-CoV-2: Severe Acute Respiratory Syndrome Coronavirus-2; SMA: smooth muscle actin; VSMC: vascular smooth muscle cell; vWF: von Willebrand factor; ↑: increase; ↓: decrease.

**Figure 2:**
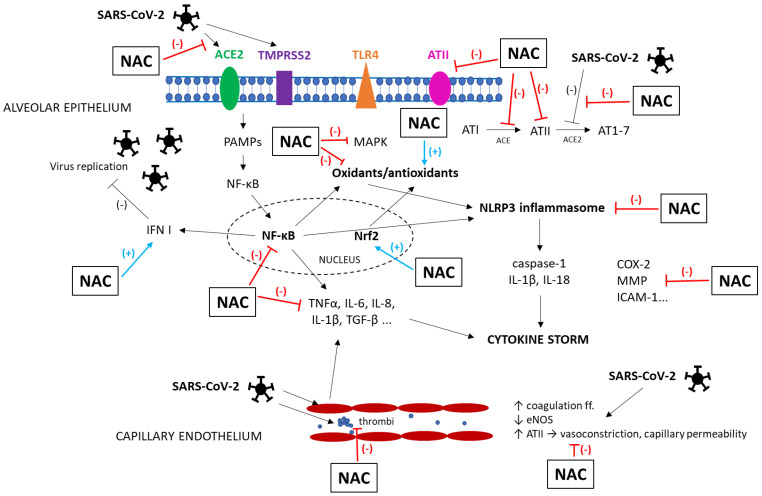
Scheme of NAC action in ARDS or COVID-19, respectively. For more details, see the text below.

In respiratory diseases, NAC may be of benefit through different mechanisms of action [55]. Other favorable effects of NAC have been discovered when it was used in COVID-19 (Figure 1 and Figure 2).

NAC is an N-acetyl derivative of the amino acid L-cysteine, which is a precursor of the highly potent antioxidant GSH. NAC administration increases GSH levels by providing cysteine for GSH synthesis in the liver, and this action contributes to redox equilibrium [56,57]. In addition, NAC also acts as a direct antioxidant, due to the content of the -SH group, which reacts with some oxidants, especially with nitrogen dioxide and hypohalous acids, and less with hydrogen peroxide and superoxide [58].

NAC likely enhances the proliferation of lymphocytes, prolongs the longevity of CD8 + cells, and decreases the production of cytokines, thereby restoring an appropriate inflammatory response in a close relation between oxidative stress and inflammation [59]. NAC also stimulates the expression of Nrf2, a transcription factor regulating antioxidant and cytoprotective enzymes [60], and via the stimulation of Nrf2, NAC suppresses inflammation and stimulates anti-inflammatory mechanisms [61]. NAC also reduces the activation of NLRP3 inflammasome, which is one of the first mechanisms of the innate immune response to viral infection [62]. Additional anti-inflammatory effects of NAC result from the suppression of oxidative stress-induced NF-κB activation [57] and also from modulation of cyclooxygenase (COX)-2, matrix metalloproteinases (MMP), mitogen-activated protein kinases (MAPK), or intercellular adhesion molecule (ICAM)-1 [63].

Moreover, the anticoagulant and thrombolytic effects of NAC could be beneficial. NAC inhibits the production of some coagulation factors (f. II, VII, IX, and X) and causes fragmentation in large von Willebrand factor (vWF) multimers by reducing disulfide bridges of sulfhydryl groups [64,65]. NAC can also reduce the activation of the coagulation cascade in severe COVID-19 [66]. Likely due to the ability of NAC to break disulfide bonds, NAC may also disrupt the aggregation of platelets and may break the bond between the blood cells and the clotting factor, thereby maintaining blood fluidity [67]. In addition, NAC has the potential to preserve endothelial function and mitigate micro-thrombosis in severe forms of COVID-19 [66]. NAC may reduce thrombotic complications by inhibiting the activity of plasminogen activator inhibitor (PAI)-1 [68], a procoagulant that positively correlated with severe cases of COVID-19 [69].

NAC protects against the harmful effects of angiotensin II, the product of ACE activity leading to vasoconstriction, inflammation, apoptosis, and generation of ROS, by inhibiting ACE activity [70,71]. The NAC-induced restoration of ACE/ACE2 balance thereby represents a valuable approach in the treatment of COVID-19.

In addition, NAC exerts its antiviral effects via activating TLR7 response to IFN-I, which is able to inhibit the replication of RNA viruses including SARS-CoV-2 [72]. NAC may also modulate the binding affinity of the SARS-CoV-2 virus to the ACE2 receptor, thus, the binding affinity is decreased when the disulfide bonds of ACE2 and SARS-CoV-2 spike proteins are reduced to sulfhydryl groups [73].

## 4. NAC in Animal Models of ALI

### 4.1. Characteristics of Animal Models of Direct and Indirect ALI

In the preclinical testing, various animal models of ALI primarily targeting the alveolar epithelium, capillary endothelium, or both alveolar epithelium and capillary endothelium can be elicited to mimic direct or indirect ARDS [74,75]. Models of direct ALI generate injury to the alveolar epithelial cells with local inflammation; elevated concentrations of cytokines (TNFα, IL-1β, IL-6, and IL-8) in the lung tissue or in the bronchoalveolar lavage fluid (BALF); alveolar edema formation; changes in surfactant proteins (SPs), particularly in SP-D, indicating damage to the type II cells; and increase in soluble RAGE (sRAGE) indicating damage to the type I cells [4,76]. These changes may be induced by, e.g., the intratracheal (i.t.) instillation of live bacteria, i.t. instillation of bacterial lipopolysaccharide (LPS), inappropriate mechanical ventilation, inhalation of toxic gases, i.t. instillation of neonatal meconium, lung contusion, or lung ischemia/reperfusion [74,75].

Models of indirect ALI cause systemic inflammation and damage to the capillary endothelium, with interstitial edema, increased plasma cytokines (IL-6 and IL-8), and vWF and angiopoietin-2 indicating endothelial damage. These changes can be elicited, e.g., by the intravenous (i.v.) or intraperitoneal (i.p.) administration of LPS, ligation, and perforation of the cecum (CLP); induction of hemorrhagic shock; ischemia/reperfusion in distant organs; i.v. instillation of oleic acid (OA); or induction of acute pancreatitis [74,75].

Epithelial/endothelial damage is also linked with a release of components of extracellular matrix (e.g., MMP) and by the activation of coagulation proven by increased PAI-1 and decreased protein C. In addition, the apoptosis of epithelial cells is increased and apoptosis of neutrophils is delayed, leading to a longer persistence of activated neutrophils at a site of injury resulting in more serious damage to the tissue [4,74,76].

### 4.2. NAC in Animal Models of Direct ALI

#### 4.2.1. NAC in a Model of Bacterial Pneumonia-Induced ALI

In the model of bacterial pneumonia, the instillation of live bacteria into the lung via the i.t. route generates pneumonia within several days. In the study by Xu et al., pretreatment with NAC (150 mg/kg/day, i.p., for 7 days) in rats before the i.t. injection of bacteria *Pseudomonas aeruginosa* mitigated lung injury and elevated eNOS protein expression, but decreased inducible NOS (iNOS) expression and did not change the bacterial loads [77].

#### 4.2.2. NAC in Models of i.t. LPS-Induced ALI

The direct airway administration of LPS by i.t. or nasal instillation or inhalation of aerosolized LPS is a common way to prepare a model of pulmonary inflammation and ALI. Depending on the used LPS dosage, robust inflammatory reaction with leukocyte infiltration, elevation of pro-inflammatory cytokines, and disruption of the alveolo-capillary barrier are induced within hours to days [74,78]. Pretreatment with NAC before i.t. LPS instillation was evaluated in several studies. For instance, Demiralay et al. compared the effects of pretreatment with different NAC doses (10, 150, 300, and 500 mg/kg) in i.t. LPS-challenged rats. While NAC at a dose of 10 mg/kg had no protective effect on the lung injury, the higher doses of NAC decreased the severity of the lung damage. However, pretreatment with NAC up to a dose of 500 mg/kg did not show any significant effect on apoptosis regulation or the local production of TNFα [79]. Pretreatment with NAC (40 mg/kg or 100 mg/kg) given by gavage 1 h before i.t LPS instillation in mice protected the lungs from oxidative stress in the first 12 h, but later no protection exerted by NAC was observed. NAC also decreased the number of inflammatory cells in the lungs and inhibited the transcription of NF-κB, IL-6, TNFα, and COX-2 induced by LPS [80]. In a more recent study, pretreatment with NAC (600 mg/kg i.g.) for 3 consecutive days with the last dose given 2 h before LPS i.t. instillation in mice significantly decreased the markers of inflammation and lung injury such as relative protein concentration, total cells, and neutrophils in the BALF; levels of IL-6, IL-8, TNFα, and IL-1β; myeloperoxidase (MPO) activity; lung injury score; and inflammatory-related gene expressions while ameliorating LPS-induced changes in antioxidative genes [81]. However, NAC may also be of benefit when given after i.t. LPS instillation. In our study, NAC administered in two different doses (10 mg/kg or 20 mg/kg i.v.) enhanced ventilatory parameters and oxygenation, decreased lung edema and leukocyte migration, and alleviated oxidative stress and inflammation, as the higher dose of NAC was more effective in reducing the oxidation of lipids and proteins, and inflammation [82].

#### 4.2.3. NAC in a Model of Ventilator-Induced ALI

Models of ventilator-induced lung injury (VILI) are produced by mechanical ventilation with high volumes and/or high pressures which trigger lung edema and inflammation [83]. In a rat model of VILI evoked by high-volume (20 mL/kg) vs. low-volume ventilation (7 mL/kg), pretreatment with NAC (140 mg/kg i.v.) given before 2 h lasting overventilation inhibited a collagen accumulation in the lungs [84].

#### 4.2.4. NAC in Models of Phosgene-Induced ALI

Exposure to toxic gases including phosgene may initially cause coughing, chest tightness, and wheezing. However, the situation may worsen within several hours to dyspnea, pulmonary edema, ARDS, or even death in severe cases [85]. Treatment with NAC (50, 100, or 200 mg/kg i.p.) after the phosgene exposure in animals alleviated lung edema and decreased oxidative markers, reversed the GSH antioxidant system, and elevated expression of Nrf2, a transcriptional factor involved in the regulation of oxidative stress, suggesting protection against oxidative stress via Nrf2/glutathione reductase/GSH pathway [60]. Contrary, treatment with NAC (approximately 350 mg given by nebulization) at 0.5, 2, 4, 6, 8, 10, and 12 h post-exposure in juvenile pigs showed no benefit for survival, improvement in oxygenation, acid-base balance, shunt fraction, lung edema, or histological score [86].

#### 4.2.5. NAC in a Model of Meconium-Induced ALI

Model of meconium aspiration syndrome is a special model mimicking neonatal ARDS due to aspiration of fetal stools (meconium). Aspirated meconium may obstruct the airways and cause dysfunction of pulmonary surfactant, triggering the generation of pulmonary edema, inflammation, oxidative stress, and vasoconstriction [83]. In experiments performed by our research group, the treatment of meconium-instilled rabbits suffering from respiratory insufficiency by NAC (10 mg/kg i.v.) significantly improved oxygenation and ventilatory parameters, decreased right-to-left pulmonary shunts, reduced lung edema, decreased total counts of cells and percentages of neutrophils and eosinophils in the BALF, diminished markers of lipid and protein oxidation in the lung homogenates and in the isolated lung mitochondria, enhanced antioxidant status, decreased eosinophil cationic protein, and decreased airway hyperreactivity [87,88]. Additional analysis showed that the NAC treatment of meconium-injured rabbits lowered plasma aldosterone, a non-specific marker of stress and injury. However, NAC i.v. delivery was associated with short-term increases in blood pressure and in several parameters of heart rate variability [89].

#### 4.2.6. NAC in Models of Lung Contusion-Induced ALI

Lung contusion is an injury to the lung parenchyma that occurs in patients with chest trauma or explosion injuries. The resulting damage to lung alveoli, capillaries, and parenchyma causes alveolar hemorrhage and parenchymal destruction [90]. In rats with moderate lung contusion, pretreatment with NAC (500 mg/kg i.v.) resulted in a slight improvement in oxygenation and a decrease in malondialdehyde (MDA) [91]. In another study, treatment with NAC (100 mg/kg i.p.) 30, 60 min, and 24 h after the damage reduced the infiltration of neutrophils and inhibited blast-induced inflammatory response [92].

#### 4.2.7. NAC in Models of Lung Ischemia/Reperfusion-Induced ALI

Ischemia followed by reperfusion, e.g., after lung transplantation may cause non-cardiogenic lung edema, inflammation, and hypoxia [74]. In a rat model of lung ischemia/reperfusion injury after the lung transplantation, NAC (150 mg/kg i.p.) was given to both donor and recipient animals 15 min before the harvest, and recipient animals were treated again before reperfusion. After 2 h of reperfusion, the NAC-treated animals showed significantly improved oxygenation, decreased lipid peroxidation, and higher levels of reduced GSH in comparison to non-treated controls [93]. In another study, NAC (50 mg/kg i.v.) treatment was given to rats before or after lung ischemia/reperfusion injury. The NAC delivery decreased nitrotyrosine (NT), cleaved caspase-3, NF-κB, TNFα, and IL-1β levels, whereas NAC given after the reperfusion potentiated the protective effects [94].

Studies demonstrating the effects of NAC in models of direct ALI are listed in Table 1.

### 4.3. NAC in Animal Models of Indirect ALI

#### 4.3.1. NAC in Models of i.p. or i.v. LPS-Induced ALI

I.p. or i.v. administration of LPS causes changes resembling sepsis, with leukopenia, lower cardiac output, arterial blood pressure, increased pulmonary artery pressure, and hypoxemia [74]. Pretreatment with NAC given as i.v. infusion (150 mg/kg/h) starting 10 min before i.v. LPS prevented LPS-induced hypotension and leukocytopenia, decreased lung damage, and lowered nitrate/nitrite, methylguanidine, TNFα, and IL-1β [95]. In another study, NAC treatment (given i.t. by nebulization or systemically by i.p. injection) was administered in mice 1 h or 22 h after i.p. LPS attenuated the hypoxic pulmonary vasoconstriction, whereas i.t. NAC given 1 h after LPS challenge was equally effective but required lower doses than systemic treatment. On the other hand, the administration of NAC 22 h after the LPS challenge did not restore the pulmonary vasoconstriction, indicating that early therapy with NAC given i.t. or i.p. may help to preserve hypoxic pulmonary vasoconstriction in sepsis-associated ALI [96]. In rats exposed to i.p. LPS, NAC (150 mg/kg) decreased the epithelial cell apoptosis and reduced the production of TNFα and VEGF, and epithelial MPO activity [97]. In another model, NAC (20 mg/kg i.p.) injected 3, 6, and 12 h after LPS i.v. injection in rats decreased lipid peroxidation in the lungs, the activity of MPO, and the concentration of NF-kB and decreased the extent of lung injury [98].

#### 4.3.2. NAC in Models of CLP-Sepsis-Induced ALI

CLP-induced peritonitis causes leukopenia, neutrophilic inflammation, interstitial and alveolar edema, hypoxemia, and pulmonary hypertension within several days [74]. In a rat model of CLP-associated ALI, the administration of NAC (4.8 g/L in drinking water) which started 2 days before sepsis induction attenuated sepsis-associated renal injury and alterations in respiratory mechanics, decreased lung edema and oxidative stress, and improved survival [99]. In another model, pretreatment with NAC (2 × 10^4^ U/kg i.v.) injected 1 h before CLP and repeated 24 h later reduced the mortality of septic rats; mitigated lung damage; reduced TNFα, IL-1β, IL-6, IL-8, and MDA; increased activity of superoxide dismutase (SOD), GSH peroxidase, and catalase; and decreased apoptosis markers in the lungs [100]. In another study, treatment with intramuscular (i.m.) NAC (150 mg/kg/day) which started 6 h after the operation and lasted for 1 week prevented increases in MPO activity and MDA, improved histopathological findings, and decreased markers of lung apoptosis [101]. In a two-hit trauma model (30% scalding burn followed by CLP 72 h later), rats were treated with NAC (150 mg/kg/day i.p.) for 72 h following CLP or were treated with NAC (150 mg/kg/day i.p.) for 6 days following thermal injury. Both treatment schemes decreased MDA levels in the liver and ileum. In addition, NAC (150 mg/kg/day i.p.) for 72 h following CLP increased GSH and decreased lung injury scores [102].

#### 4.3.3. NAC in Models of Hemorrhagic Shock-Induced ALI

Hemorrhagic shock in trauma may be modeled by the pressure-controlled withdrawal of the blood followed by transfusion of the removed blood and lactate Ringer solution [103]. In rats with hemorrhagic shock, pretreatment with NAC (0.5 mg of NAC/dL of drink water) did not lead to any obvious differences in lipid peroxidation markers or GSH levels in the lungs or serum [104]. In another study, treatment with NAC (150 mg/kg/h i.v.) starting 15 min after the insult improved histopathologic scores in both lungs and kidneys, lowered lung and kidney MDA levels, serum nitrite/nitrate and IL-6, and decreased NF-κB p65 DNA binding activity [105]. The addition of NAC (150 mg/kg) in resuscitation Ringer’s lactate decreased cell counts in the BALF, infiltration of lungs, and MDA [106].

#### 4.3.4. NAC in a Model of Renal Ischemia/Reperfusion-Induced ALI

Ischemia followed by reperfusion even in distant vascular beds can result in lung injury [74]. In a rat model of ALI associated with acute kidney injury (AKI) due to renal ischemia/reperfusion, pretreatment with NAC (150 mg/kg or 500 mg/kg i.p.) for 3 days started 2 h before induction of AKI. NAC (150 mg/kg) decreased the serum creatinine; however, NAC did not improve kidney damage. On the other hand, this dose of NAC diminished lung injury score, although no differences were observed in the lung edema, endothelial permeability, or serum levels of MDA and nitrite [107].

#### 4.3.5. NAC in Models of OA-Induced ALI

The model of OA-induced lung injury mimics pulmonary lipid embolism in patients with long bone trauma. I.v. delivery of OA causes fast damage to endothelial cells followed by a lung epithelial injury and edema with serious ventilation/perfusion mismatch [74]. In OA-induced ALI, pretreatment with NAC (150 mg/kg i.v.) was given to rats 15 min before i.v. OA infusion or 2 h after OA infusion reduced tissue MPO, MDA, and 3-NT levels compared to non-treated animals [108]. In another study, pretreatment with NAC (163 mg/kg i.p.) before i.v. OA instillation enhanced respiratory and cardiac performance and prolonged survival; however, failed to prevent the development of lung edema [109].

#### 4.3.6. NAC in a Model of Acute Pancreatitis-Induced ALI

ARDS is a common systemic complication of acute pancreatitis. Released inflammatory mediators damage not only the local peripancreatic tissue but may cause a systemic inflammatory response and multiple organ dysfunction syndrome [110]. In the study by Yubero et al. [111], rats received NAC (50 mg/kg i.p.) 1 h before inducing pancreatitis followed by a new injection 1 h afterward. Although NAC treatment reduced the production of inflammatory mediators in the lungs, it did not prevent leukocyte infiltration [111].

Studies demonstrating the effects of NAC in models of indirect ALI are listed in Table 2.

## 5. NAC in Patients with ARDS

NAC has been used in numerous clinical trials as well. For instance, treatment with NAC (40 mg/kg/d i.v., n = 32) or placebo instead of NAC in controls (n = 29) was given for 3 days in mechanically ventilated patients presenting with mild-to-moderate ARDS due to various underlying diseases. NAC treatment significantly improved systemic oxygenation during 3 days, caused a regression of lung injury score (LIS) during the first 10 days of treatment, and reduced the need for ventilatory support. In addition, no NAC-associated adverse effects were observed [112].

In the randomized, double-blind, placebo-controlled, prospective clinical trial, patients with ARDS requiring mechanical ventilation were given standard care for ARDS and i.v. infusion of NAC (70 mg/kg, n = 14), or procysteine (L-2-oxothiazolidine-4-carboxylate, OTZ, 63 mg/kg, n = 17), or placebo (n = 15) every 8 h for 10 days. Treatment with both antioxidants repleted the GSH levels in red blood cells over the treatment period. Although there was no difference in mortality among groups, both treatments resulted in a lower number of days with ARDS and a higher cardiac index [113].

In a randomized, double-blind, placebo-controlled clinical study by Dominghetti et al., patients with an established ARDS subsequent to a variety of underlying diseases were treated with either NAC (190 mg/kg/day, n = 22) or placebo (n = 20) as a continuous i.v. infusion over the first 3 days of their clinical course. Except for decreased LIS, NAC treatment during 72 h neither improved systemic oxygenation nor reduced the need for ventilatory support [114].

In a randomized controlled trial by Soltan-Sharifi et al., 17 ARDS patients received NAC (150 mg/kg i.v. on the first day followed by 50 mg/kg/day for 3 days) and 10 patients obtained the standard therapy without NAC. Treatment with NAC increased extracellular total antioxidant power, total thiol molecules, and intracellular GSH which were associated with improved outcomes for the patients [115].

In a randomized controlled trial by Zhang et al., patients with community-acquired pneumonia received conventional treatment (n = 24 of NAC-nontreated group) or conventional treatment plus NAC (600 mg tablets, a dose of 1200 mg/d p.o., for 10 days, n = 37). NAC treatment led to a significant decrease in plasma MDA and TNFα and an increase in total antioxidant capacity compared to the NAC-nontreated group, while no NAC-related adverse effects were observed. However, the addition of NAC into a treatment protocol did not influence plasma SOD activity or computer tomography score [116].

In another randomized controlled trial, NAC was evaluated as a tool for the prevention of ventilator-associated pneumonia (VAP). In mechanically ventilated patients hospitalized in ICU at high risk of developing VAP (n = 30), NAC (600 mg) was given twice daily via nasogastric tube in addition to routine care. NAC pretreatment was associated with a lower likelihood of developing clinically confirmed VAP compared with patients treated with placebo, more patients reached complete recovery and the NAC-treated patients spent less time in ICU than the patients receiving placebo [117].

In a recent randomized clinical trial, mechanically ventilated patients with ARDS were treated with NAC (150 mg/kg on the first day of admission and then 50 mg/kg up to the fourth day of admission, n = 30), while patients in the control group (n = 30) received routine care without NAC. There were no significant differences in the duration of hospitalization in the ICU, time required for mechanical ventilation, or mortality rate of the patients in the NAC-treated vs. control groups. The authors found no between-group differences in mean arterial blood pressure, heart rate, respiratory rate, oxygen saturation, APACHE II score, or pulmonary capacity in the first four days after the intervention. However, there was a significant difference in the level of consciousness, PaO_2_/FiO_2_ index, and PEEP of the NAC-treated patients vs. controls within 3 to 4 days after the intervention [118].

Studies demonstrating the effects of NAC in clinical trials of ARDS are listed in Table 3.

## 6. NAC in Patients with COVID-19

During the COVID-19 pandemic and in the period after its end, several articles provided information on the experience with NAC treatment in the treatment of this disease [59,67,119,120,121,122]. Several clinical studies showed an improvement in the health status of the NAC-treated patients and a reduction in markers of inflammation and oxidative stress, with a more pronounced effect observed in patients with a more severe course of the disease. For example, Ibrahim et al. reported an improved condition in 10 mechanically ventilated patients with COVID-19, including one patient with glucose-6-phosphate dehydrogenase (G6PD) deficiency treated with hydroxychloroquine whose treatment with NAC lasted from 2 to 9 days. The administration of NAC (30,000 mg i.v. in 2 days) alleviated hemolysis and decreased COVID-19-associated increases in liver enzymes, C-reactive protein (CRP), and ferritin in the G6PD-deficient patient. In the additional 9 patients, NAC treatment (20,000 mg given in 2 days in one patient, 600 mg given every 12 h in an additional 8 patients) improved the clinical status and reduced inflammatory markers (CRP and ferritin). Finally, 8 patients were discharged from the hospital, and the remaining 2 patients showed significant clinical improvement [123].

In a single-center, double-blind, randomized, placebo-controlled trial conducted in Brazil, n = 135 patients with severe COVID-19 were given NAC in a single dose of 21 g (300 mg/kg) for 20 h. Treatment with a high-dose NAC did not affect the course of the disease and no differences were observed in mortality, duration of mechanical ventilation, or need for ICU admission compared to the control group [124].

Similarly, a study by Taher et al., where patients with mild to moderate COVID-19 (n = 47) were given NAC (40 mg/kg/day i.v.) for 3 consecutive days in addition to standard treatment, showed a slight improvement in the patients’ condition, but did not result in significant differences in 28-day mortality, need for invasive ventilatory support, number of days with ventilatory support, number of days spent in the ICU, or length of hospital stay, nor differences in oxygenation or the Sequential Organ Failure Assessment (SOFA) score compared to the control group [125].

In the large study by Faverio et al., patients with COVID-19 (n = 585) were treated with NAC for at least 5 days, initially at a dose of 300 mg i.v. 3 times a day, which was reduced to 600 mg p.o. twice a day after the patient’s condition stabilized. In the surviving patients of the study (n = 102), data on the development of their health condition, including lung function tests, X-rays, and performance tests, were recorded for 6 months after discharge from the hospital. The authors found that patients treated with NAC had shorter hospitalization periods than patients not treated with NAC. However, no differences were observed in other parameters (ICU admission or in-hospital mortality) between the NAC-treated and NAC-untreated patients [126].

Compared to the above-mentioned studies with a short administration period [124,125], more favorable results were demonstrated with longer delivery of NAC. For instance, in the study by Avdeev et al., patients with COVID-19 (n = 24) were treated with NAC at a daily dose of 1200–1800 mg i.v. After 10 days, significant improvements in oxygenation, CRP, and the National Early Warning Score (NEWS)2 were observed compared to the control group. Other endpoints (transfer to ICU, need for non-invasive or invasive mechanical ventilation, or 28-day mortality) did not change compared to the control group. The authors did not observe any adverse events related to the administration or discontinuation of NAC treatment [127].

In a retrospective study by Assimakopoulos et al., the addition of NAC (600 mg p.o. twice a day) to standard treatment for 14 days in patients with moderate to severe COVID-19 (n = 42) slowed down the disease progression compared to the control group and reduced the mortality after 14 and 28 days, especially in patients with severe disease. NAC improved oxygenation, reduced the number of leukocytes in the blood, and reduced levels of CRP, D-dimers, and lactate dehydrogenase. In addition, the NAC treatment reduced the need for mechanical ventilation and mortality [128].

In a large observational retrospective cohort study of hospitalized COVID-19 patients in Spain, NAC administered orally (600 mg every 8 h) was added to standard therapy (in n = 2071 patients). NAC-treated COVID-19 patients were older, predominantly male, and with more comorbidities when compared with those not treated with NAC. Despite greater baseline risk, NAC administration was associated with significantly lower mortality although there were no significant effects on the mean duration of hospitalization, ICU admission, or use of invasive mechanical ventilation [129].

Chavarría et al. presented the results of a study where patients with COVID-19 were treated with a combination of an antioxidant (vitamin C, D, or E; NAC; or melatonin) with pentoxifylline. Oral administration of NAC (600 mg effervescent tablets twice daily) for 5 days in combination with pentoxifylline reduced lipid peroxidation, IL-6, CRP, and procalcitonin levels and increased total antioxidant capacity and plasma nitrite levels in patients with both mild and severe COVID-19 more significantly than after the treatment with the pentoxifylline-only [130].

In a randomized controlled trial in n = 30 patients with COVID-19, Atefi et al. evaluated the efficacy and safety of oral NAC treatment (600 mg) added to routinely administered antivirals and hydroxychloroquine. The authors found that the groups with added NAC had reduced mortality, decreased CRP, and higher oxygen saturation [131].

In the randomized controlled clinical trial study conducted on patients with COVID-19, n = 250 patients were randomly allocated into the intervention group (routine treatment + NAC inhaler spray, one puff per 12 h, for 7 days) or the control group in which the patients received routine treatment without NAC. NAC treatment significantly decreased mortality rate, white blood cell count, CRP, and aspartate aminotransferase, but this treatment had no effect on length of hospital stay or need of ICU admission [132].

Studies demonstrating the effects of NAC in trials of COVID-19 are listed in Table 4.

In addition, there have been several randomized controlled trials performed which investigated the use of NAC in COVID-19 [67]. For instance, the NCT04374461 trial has included patients with severe COVID-19 infections who have received NAC 6 g/day i.v. in addition to supportive and/or COVID-19-directed treatments at the discretion of the treating physician. The primary outcome measures have included the number of patients who were successfully extubated and/or transferred out of critical care due to clinical improvement and that of patients who were discharged from the hospital due to clinical improvement (study completion has been estimated to 2025). The NCT04792021 trial has evaluated the effect of NAC (600 mg orally) on oxidative stress and the occurrence of complications in COVID-19 patients based on the change in TNFα. The other NCT04455243 trial is planned to evaluate the efficacy of oral/i.v. NAC therapy (150 mg/kg every 12 h for 14 days) in the management of adult patients with COVID-19 according to time to recovery as the primary outcome measure. The NCT04419025 trial is planned to estimate the efficacy of oral NAC in preventing COVID-19 from progressing to severe disease. However, no data from these trials have been published up to now.

Summarizing their experience with the use of NAC in COVID-19, Shi and Puyo [133] proposed the treatment options and dosages of NAC in COVID-19 based on clinical observation and evidence: as prevention for health care workers or people at high risk with comorbidities—NAC 1200 mg/day; for a mild disease—to double up the dose; for moderate to severe symptoms—NAC 100 mg/kg/day i.v. for 7 to 10 days upon hospital admission; for severe to critically ill—NAC 150 mg/kg/day i.v. for 7 to 10 days upon hospital admission. The authors assumed that when a higher concentration of intravenous NAC is given, better clinical outcomes could be expected because of the effective reduction in viral replication and significant alleviation of pneumocyte damage, as well as the modulation of immune responses and therefore prevention of a “cytokine storm” [133].

Positive response to NAC treatment in COVID-19 patients was also shown in several case reports. For instance, significant improvement in health condition was observed in a 45-year-old patient with severe SARS-CoV-2 pneumonia and several serious comorbidities, in whom the infusion of high-dose NAC (NAC 10 g in 500 mL of 5% dextrose solution at 21 mL/h for 2 days) reduced levels of CRP and procalcitonin, and enabled to decrease FiO_2_ and PEEP. When the patient’s antibodies against SARS-CoV-2 remained negative, he received a maintenance infusion treatment with NAC (NAC at 2.5 g in 250 mL 5% dextrose solution infused over 4 h twice daily (100 mg/kg/day) for the next 3 weeks. Ten days later, the patient was discharged home [134]. NAC was also effective in patients with cancer who were affected by COVID-19. For instance, Liu et al. demonstrated that BALF by using NAC inhalation solution significantly enhanced airway clearance and reduced refractory hypercapnia in a 64-year-old patient with an anastomotic fistula after the radical treatment of esophageal cancer and right-side encapsulated pyopneumothorax [135]. In a subset of COVID-infected gynecological cancer patients, treatment with NAC (oral 600–1200 mg, twice a day) in three patients vs. six patients as NAC-nontreated controls improved subjective shortness of breath, brain fog, and fatigue, and normalized vWF levels [136]. In another case study, Carothers et al. reported two cases of suspected remdesivir-associated acute liver failure in which the liver failure was alleviated after continuous NAC infusion and withdrawal of remdesivir. Remdesivir as a nucleoside RNA polymerase inhibitor used in the treatment of COVID-19 pneumonia caused in both patients a significant increase in transaminases which was accompanied by coagulopathy and encephalopathy. Continuous infusion of NAC (150 mg/kg over 1 h, 50 mg/kg over 4 h, and 100 mg/kg over 16 h) within 12 h decreased levels of transaminases in both patients [137].

Nevertheless, a recent meta-analysis of five randomized controlled trials showed no significant differences in mortality, length of hospital stay, need for ICU admission, length of ICU stay, or use of invasive mechanical ventilation between the patients treated with NAC and those NAC-nontreated [122].

## 7. Limitations and Future Challenges

Considering the previously mentioned findings, inconsistency in experience with NAC between animal studies and clinical trials can be observed. The results from the animal studies are relatively homogenous because of keeping any standard methods including delivery of the treatment that are obligatory for all animals included in the study. On the other hand, clinical studies are largely heterogeneous because of diversity in numerous factors, e.g., different dosages and ways of administration of NAC or eventually any co-administered treatments; inclusion/exclusion criteria of the study; and composition of the groups of patients according to their age, gender, subtype of ARDS, and dysfunction of remote organs. These differences are likely responsible for the situation that although several trials have shown an improvement in some indices, results of large clinical meta-analyses are not able to undoubtedly show significantly reduced mortality or other benefits such as a lower need for mechanical ventilation or shorter ICU stay [48,138,139].

In addition, proper administration of antioxidants including NAC faces several limitations because of their biochemical, biophysical, and biological properties, such as low permeability into the cells, low bioavailability linked with insolubility or instability, interactions with barriers in the gastrointestinal system (low pH of gastric juice, interaction with intestinal mucosal lining, selective permeability of enterocyte membranes, etc.), and relatively fast metabolism [39,139,140].

Several possibilities have arisen to enhance the effectiveness of NAC treatment. Better results could be observed if NAC is administered for a longer time, e.g., 7–10 days, as demonstrated in several studies on NAC treatment in ARDS [113,116,117] or COVID-19 [67,133]. The effect of NAC therapy could also be enhanced by the use of high doses of the drug. However, excessive doses of antioxidants may bring no additional benefits or may even be deleterious, as it was previously demonstrated, e.g., for high doses of vitamin E or vitamin C [141]. Although the overproduction of ROS has various negative effects on the body, free radicals actively participate in the immune responses including the elimination of pathogenic microbes, and thus, excessive use of exogenous antioxidants may be harmful because of inducing oxidation–antioxidant imbalance [128,142].

Rather high variation in plasma concentrations following the oral administration of NAC [143] may be ameliorated by the inhalation route of administration [132,144,145]. However, multiple airway branching and unique pulmonary barriers cause the drug delivery efficiency to the lungs to be relatively low and may be associated with undesired side effects [146]. In addition, data on the effectiveness of nebulized or spray delivery of NAC in ARDS patients are limited.

Other promising approach potentially increasing the effectiveness of NAC is a combination with other antioxidants or other drugs mitigating inflammation and lung damage. For instance, the combination of two antioxidants, NAC and deferoxamine, improved survival and effectively decreased neutrophil infiltration and oxidative stress in the organs involved in septic response in a rat model of CLP-induced sepsis [147]. In the following study on the i.t. LPS-induced rat model of ALI, this treatment combination significantly reduced histopathologic alterations, lung edema, inflammation, and markers of oxidative damage more effectively than these treatments given individually [148].

However, NAC may be easily combined with other drugs, as well. The results from our research group show that the combined use of i.v. NAC with i.t. exogenous surfactant significantly improved lung functions, alleviated lung inflammation and ventilation-perfusion mismatch, and decreased oxidative stress markers and edema in a double-hit rat model of ALI due to i.t. instillation of LPS followed by hyperoxia [149] as well as in a rabbit model of meconium-induced ALI [150,151,152]. The combination of NAC with corticosteroid dexamethasone potentiated a protective effect on AW hyperresponsiveness and inflammation caused by chlorine inhalation-induced ALI in mice [153]. In a case study by Martini et al., the combination of NAC with steroids also improved the clinical outcome of the patient with COVID-19-induced autoimmune hepatitis [154]. In a rat model of pancreatitis, pretreatment with crotapotin, a phospholipase A_2_ inhibitor, plus NAC effectively prevented both pulmonary morphological and mechanical changes induced by acute pancreatitis [155]. Similarly, pretreatment with NAC plus atorvastatin for 3 days before mesenteric ischemia/reperfusion reduced the associated inflammation and lung injury more effectively than their individual use [156]. Another very effective combination was the use of NAC and dexmedetomidine, a sympatholytic drug acting as an agonist of α2-adrenergic receptors. In a murine LPS-induced model of ALI, NAC and dexmedetomidine attenuated the lung morphological damage, edema, cell infiltration, aberrant MPO activity, and production of Th1/Th2/Th17 cytokines, with the most potent effect found for NAC plus dexmedetomidine combination [157]. The favorable potential of the combination of NAC with other drugs was also confirmed by several clinical case reports, as well. For instance, the combination of nebulized NAC (20% NAC, 3 mL every 4 h) with nebulized albuterol sulfate and aerosolized heparin significantly improved the pulmonary status of a 47-year-old patient with severe chlorine-induced ARDS [158]. Similarly, the concomitant administration of three nebulized drugs, NAC (600 mg/3 mL, every 4 h), heparin, and epoprostenol, effectively enhanced the lung functions in a 24-year-old patient with a smoke inhalational injury and burn-associated ARDS [159]. More recently, a combination of nebulized NAC (5 mL of 10% NAC every 6 h) and heparin significantly enhanced the clinical status of a patient with inhalational injury and burn and prevented the development of ARDS [160].

Moreover, the efficacy of NAC may be enhanced by novel drug-delivery approaches [139]. Nanocarriers may increase the stability of antioxidants upon encapsulation and thereby improve the transport into the cells compared with free antioxidant compounds [39]. For example, NAC loaded in a biocompatible porous silica Nano protected against LPS-induced ALI through anti-oxidative and anti-inflammatory effects, which were superior to those of NAC-only [161]. Similarly, pretreatment with liposomally entrapped NAC more effectively prevented LPS-induced lung injuries than NAC-only [162,163]. Another perspective possibility of the use of NAC is its conjugation with S-allyl mercaptan, an active ingredient of allicin, forming S-allylmercapto-N-acetylcysteine. This conjugate exerted more potent inhibition of inflammation and oxidative stress in a murine LPS-induced model of ALI than NAC-only [164].

## 8. Conclusions

Multiple mechanisms of action which cover scavenging ROS, replenishing intracellular GSH, mitigating inflammation and coagulation, suppressing cytokine storm, and suppressing SARS-CoV-2 replication propose the use of NAC in the treatment of various forms of ARDS including COVID-19. In spite of favorable findings from animal studies, the results of clinical trials are rather heterogeneous. While several studies have confirmed an improvement in the condition of patients after NAC treatment, shortening the duration of hospitalization, and a trend to reduced mortality, other studies failed to confirm this effect. The discrepancy likely results from heterogeneity in dosage and the way of administration of NAC, inclusion/exclusion criteria of the trials, composition of the groups of patients, subtype of ARDS, etc. From this reason, large-scale multicenter trials should be conducted to obtain credible results. Additional future challenges deal with solving the biological limitations of NAC which may be at least partially overcome by introducing novel drug-delivery approaches or appropriate combinations of NAC with other drugs. In conclusion, although NAC is relatively safe, cost-effective, widely available, and has been FDA-approved, due to limited evidence currently available, it is premature to formulate any general recommendations regarding the use of NAC in ARDS before the outcomes of the ongoing clinical trials are published.

## Figures and Tables

**Table 1:** Models of direct ALI with NAC as a post-exposure treatment (more details are provided in the text).

Triggering Factor	Species	NAC Dose/Way of Delivery	Major Findings	Ref.
I.t. LPS	Wistar rats	NAC (10 mg/kg or 20 mg/kg i.v.) after elicitation of respiratory insufficiency	↑ ventilatory parameters and oxygenation, ↓ lung edema, ↓ oxidative stress, ↓ inflammation	[82]
Phosgene inhalation	SD rats	NAC (50, 100, or 200 mg/kg i.p.) after exposure to phosgene	↓ lung edema, ↓ markers of oxidative stress, ↑ Nrf2	[60]
Phosgene inhalation	Juvenile pigs	NAC (cca 350 mg) given by nebulization 0.5, 2, 4, 6, 8, 10, and 12 h post-exposure	No improvement in survival, gas exchange, shunt fraction, lung edema, or histological score	[86]
Meconium instillation	Rabbits	NAC (10 mg/kg i.v.) 30 min after the induction of respiratory insufficiency	↑ oxygenation, ↓ right-to-left pulmonary shunts, ↓ lung edema, ↓ oxidative stress, ↓ inflammation, and ↓ AW hyperreactivity	[87,88]
Lung contusion	SD rats	NAC (100 mg/kg i.p.) 30, 60 min, and 24 h after the blast damage	↓ neutrophil infiltration of lungs and ↓blast-induced inflammatory response	[92]
Lung ischemia/reperfusion	Wistar rats	NAC (50 mg/kg i.v.) treatment before or after the insult	↓ nitrotyrosine, cleaved caspase-3, NF-κB, TNFα, and IL-1β levels	[94]

Abbreviations: ALI: acute lung injury; AW: airway; IL: interleukin; i.p.: intraperitoneal administration; i.t.: intratracheal administration; i.v.: intravenous administration; LPS: lipopolysaccharide; NAC: N-acetylcysteine; NF-κB: nuclear factor-kappa B; Nrf2: nuclear factor erythroid 2-related factor 2; SD rats: Sprague Dawley rats; TNFα: tumor necrosis factor-alpha; ↑: increase; ↓: decrease.

**Table 2:** Models of indirect ALI with NAC as a post-exposure treatment (more details are provided in the text).

Triggering Factor	Species	NAC Dose/Way of Delivery	Major Findings	Ref.
I.p. LPS	SV129/B6F1 mice	NAC (500 mg/kg i.p. or 100 mg/kg i.t. by aerosol) given 1 h and 22 h after i.p. LPS challenge	Early delivery of i.p. or i.t. NAC ↓ hypoxic pulmonary vasoconstriction	[96]
I.p. LPS	Wistar rats	NAC (150 mg/kg p.o.) following i.p. LPS	↓ apoptosis of epithelial lung cells and ↓ TNFα, VEGF, and MPO	[97]
I.v. LPS	SD rats	NAC (20 mg/kg i.p.) given 3, 6, and 12 h after LPS i.v. injection	↓ lipid peroxidation in the lung, ↓ MPO activity, ↓ NF-kB, and ↓ extent of lung injury	[98]
CLP sepsis	Wistar rats	NAC (150 mg/kg/day i.m.) initiated 6 h after operations, for 1 week	↓ MPO activity, ↓ MDA in lung, improved histopathology, and ↓ apoptosis	[101]
Two-hit (burn + CLP) insult	Wistar rats	NAC (150 mg/kg/day i.p.), 72 h after CLP	↓ MDA in liver and ileum, ↑ lung GSH, and ↓ lung injury score	[102]
Hemorrhagic shock	SD rats	NAC (150 mg/kg/h i.v.) initiated 15 min after the insult	↓ MDA, ↓ nitrite/nitrate, ↓ IL-6, ↓ NF-κB p65 DNA activity, and improved histopathology	[105]
Hemorrhagic shock	Wistar rats	Addition of NAC (150 mg/kg) in resuscitation Ringer’s lactate solution	↓ cell counts in BALF, ↓ MDA, and ↓ inflammatory infiltration	[106]

Abbreviations: ALI: acute lung injury; BALF: bronchoalveolar lavage fluid; CLP: cecal ligature and puncture; DNA: deoxyribonucleic acid; GSH: glutathione; IL: interleukin; i.m.: intramuscular administration; i.p.: intraperitoneal administration; i.t.: intratracheal administration; i.v.: intravenous administration; LPS: lipopolysaccharide; MDA: malondialdehyde; MPO: myeloperoxidase; NAC: N-acetylcysteine; NF-κB: nuclear factor-kappa B; p.o.: (per)oral administration; SD rats: Sprague Dawley rats; TNFα: tumor necrosis factor-alpha; VEGF: vascular endothelial growth factor; ↑: increase; ↓: decrease.

**Table 3:** Clinical studies demonstrating effects of NAC in ARDS. For more details, see the text.

Subtype of ARDS	No. of Patients	NAC Dose/Way of Delivery	Major Findings/Outcomes	Ref.
Mild-to-moderate ARDS	NAC n = 32, placebo n = 29	NAC (40 mg/kg/d i.v.) or placebo in controls, given for 3 days	↑ oxygenation, regression of lung injury score, ↓ need for ventilation, and no adverse effects	[112]
ARDS requiring mechanical ventilation	NAC n = 14, OTZ n = 17, placebo n = 15	NAC (70 mg/kg) or procystein (OTZ, 63 mg/kg), or placebo given by i.v. infusion every 8 h for 10 days	Repletion of GSH in red blood cells, ↑ cardiac index, and ↓ number of days with ARDS	[113]
ARDS requiring mechanical ventilation	NAC n = 22, placebo n = 20	NAC (190 mg/kg/d) or placebo, continuous i.v. infusion, for the first 3 days	↓ lung injury score, no improvement in oxygenation, and no reduction in the need for ventilation	[114]
ARDS requiring mechanical ventilation	NAC n = 17, NAC-nontreated n = 10	NAC (150 mg/kg i.v. on the first day followed by 50 mg/kg/day for 3 days) and controls obtained the standard therapy	↑ extracellular total antioxidant power, ↑ total thiols, ↑ GSH, and improved outcome	[115]
Community-acquired pneumonia	NAC n = 37, NAC-nontreated n = 24	NAC (600 mg tablets, a dose of 1200 mg/d p.o., for 10 days) + conventional therapy and controls treated by conventional therapy	↓ plasma MDA and TNFα, ↑ total antioxidant capacity, no effect on SOD, and no improvement in CT	[116]
Ventilator-associated pneumonia	NAC n = 30, NAC-nontreated n = 30	NAC (600 mg) given twice daily via nasogastric tube in addition to routine care	↓ development of clinically confirmed pneumonia, shorter stay in ICU, and more patients with complete recovery	[117]
ARDS requiring mechanical ventilation	NAC n = 30, NAC-nontreated n = 30	NAC (150 mg/kg on the day 1 of admission, then 50 mg/kg up to day 4 of admission) and control group given routine care without NAC	Improved level of consciousness, oxygenation, and PEEP within 3–4 days of intervention	[118]

Abbreviations: ARDS: acute respiratory distress syndrome; CT: computer tomography; GSH: glutathione; ICU: intensive care unit; i.v.: intravenous administration; MDA: malondialdehyde; NAC: N-acetylcysteine; OTZ: L-2-oxothiazolidine-4-carboxylate; PEEP: positive end-expiratory pressure; p.o.: (per)oral administration; SOD: superoxide dismutase; TNFα: tumor necrosis factor-alpha; ↑: increase; ↓: decrease.

**Table 4:** Clinical studies demonstrating effects of NAC in COVID-19. For more details, see the text.

No. of Patients	NAC Dose/Way of Delivery	Major Findings/Outcomes	Ref.
n = 10	One G6PD-deficient patient: 30,000 mg i.v. NAC in 2 days, one patient: 20,000 mg i.v. NAC in 2 days, and additional eight patients: 600 mg i.v. NAC every 12 h	↓ liver enzymes, CRP, and ferritin in G6PD-deficient patient; improved clinical status; and ↓ inflammatory markers (CRP and ferritin)	[123]
n = 135	NAC in a single dose of 21 g (300 mg/kg) for 20 h	No significant improvement in mortality, duration of mechanical ventilation, or need for ICU admission	[124]
n = 47	NAC (40 mg/kg/day i.v.) for 3 consecutive days in addition to standard treatment	Slight improvement in the patients’ condition, but no differences in mortality, need for ventilation, or hospital stay	[125]
n = 585	NAC for at least 5 days, initially a dose of 300 mg i.v. three times a day, then reduced to 600 mg p.o. twice a day after the patient’s condition stabilized	Shorter hospitalization period, but no differences in mortality or ICU admission	[126]
n = 24	NAC i.v. at a daily dose of 1200–1800 mg	Improvements in oxygenation, CRP, and NEWS2 score, but no differences in mortality, need for ventilation or ICU admission	[127]
n = 42	NAC (600 mg p.o. twice a day) to a standard treatment for 14 days	↓ disease progression, ↑ oxygenation, ↓ blood leukocytes, ↓ CRP, D-dimers, LDH, and ↓ mortality and need for ventilation	[128]
n = 2071	NAC (600 mg p.o. every 8 h) added to standard therapy	↓ mortality, but no differences in need for ventilation, ICU admission, or duration of hospitalization	[129]
n = 125	NAC inhaler spray (one puff per 12 h, for 7 days) + routine treatment	↓ mortality, ↓ leukocyte count, ↓ CRP, and ↓ AST, but no differences in ICU admission or duration of hospitalization	[132]

Abbreviations: AST: aspartate aminotransferase; CRP: C-reactive protein; G6PD: glucose-6-phosphate dehydrogenase; ICU: intensive care unit; i.v.: intravenous administration; LDH: lactate dehydrogenase; NAC: N-acetylcysteine; NEWS2: the National Early Warning Score 2; p.o.: (per)oral administration; ↑: increase; ↓: decrease.

## Data Availability

Data are available within the article.

## Abbreviations

The following abbreviations are used in this manuscript:

ACE	angiotensin-converting enzyme
AKI	acute kidney injury
ALI	acute lung injury
ARDS	acute respiratory distress syndrome
BALF	bronchoalveolar lavage fluid
CLP	ligation and perforation of the cecum
COVID-19	Coronavirus disease 2019
COX-2	cyclooxygenase-2
CRP	C-reactive protein
eNOS	Endothelial nitric oxide synthase
FiO_2_	fraction of inspired oxygen
G6PD	glucose-6-phosphate dehydrogenase
GSH	glutathione
i.m.	intramuscular
i.p.	intraperitoneal
i.t.	intratracheal
i.v.	intravenous
ICAM-1	intercellular adhesion molecule-1
ICU	intensive care unit
IFN	interferon
IL	interleukin
iNOS	Inducible nitric oxide synthase
LIS	lung injury score
LPS	lipopolysaccharide
MAPK	mitogen-activated protein kinase
MCP	monocyte chemoattractant protein
MDA	malondialdehyde
MMP	matrix metalloproteinases
MPO	myeloperoxidase
NAC	N-acetylcysteine
NADPH	nicotinamide adenine dinucleotide phosphate
NETs	neutrophil extracellular traps
NF-κB	nuclear factor-kappa B
NLRP3	NLR family pyrin domain containing 3
NO	nitric oxide
Nrf2	nuclear factor erythroid-derived 2-like 2
NT	nitrotyrosine
OA	oleic acid
OTZ	L-2-oxothiazolidine-4-carboxylate
p.o.	(per)oral
PAF	platelet-activating factor
PAI-1	plasminogen activator inhibitor-1
PAMPs	pathogen-associated molecular patterns
PaO_2_	arterial partial pressure of oxygen
PEEP	positive end-expiratory pressure
RAGE	receptor for advanced glycation end products
RNA	Ribonucleic acid
ROS	reactive oxygen species
SARS-CoV-2	Severe Acute Respiratory Syndrome Coronavirus-2
SOD	superoxide dismutase
SP	surfactant protein
TGF-β	transforming growth factor-beta
TLR	Toll-like receptor
TMPRSS	transmembrane protease serine
TNFα	tumor necrosis factor-alpha
VAP	ventilator-associated pneumonia
VEGF	vascular endothelial growth factor
VILI	ventilator-induced lung injury
vWF	von Willebrand factor

## References

[1] Ashbaugh D.G., Bigelow D.B., Petty T.L., Levine B.E. (1967). Acute respiratory distress in adults. Lancet.

[2] Bernard G.R., Artigas A., Brigham K.L., Carlet J., Falke K., Hudson L., Lamy M., Legall J.R., Morris A., Spragg R. (1994). The American-European Consensus Conference on ARDS. Definitions, mechanisms, relevant outcomes, and clinical trial coordination. Am. J. Respir. Crit. Care Med..

[3] Ranieri V.M., Rubenfeld G.D., Thompson B.T., Ferguson N.D., Caldwell E., Fan E., Camporota L., Slutsky A.S., ARDS Definition Task Force (2012). Acute respiratory distress syndrome: The Berlin Definition. J. Am. Med. Assoc..

[4] Mokrá D. (2020). Acute lung injury—From pathophysiology to treatment. Physiol. Res..

[5] Seeley E.J. (2013). Updates in the management of acute lung injury: A focus on the overlap between AKI and ARDS. Adv. Chronic Kidney Dis..

[6] Bellani G., Laffey J.G., Pham T., Fan E., Brochard L., Esteban A., Gattinoni L., van Haren F., Larsson A., McAuley D.F. (2016). Epidemiology, Patterns of Care, and Mortality for Patients with Acute Respiratory Distress Syndrome in Intensive Care Units in 50 Countries. J. Am. Med. Assoc..

[7] Auriemma C.L., Zhuo H., Delucchi K., Deiss T., Liu T., Jauregui A., Ke S., Vessel K., Lippi M., Seeley E. (2020). Acute respiratory distress syndrome-attributable mortality in critically ill patients with sepsis. Intensive Care Med..

[8] Meyer N.J., Gattinoni L., Calfee C.S. (2021). Acute respiratory distress syndrome. Lancet.

[9] Herridge M.S., Moss M., Hough C.L., Hopkins R.O., Rice T.W., Bienvenu O.J., Azoulay E. (2016). Recovery and outcomes after the acute respiratory distress syndrome (ARDS) in patients and their family caregivers. Intensive Care Med..

[10] Bein T., Weber-Carstens S., Apfelbacher C. (2018). Long-term outcome after the acute respiratory distress syndrome: Different from general critical illness?. Curr. Opin. Crit. Care.

[11] Ware L.B., Matthay M.A. (2000). The acute respiratory distress syndrome. N. Engl. J. Med..

[12] Spadaro S., Park M., Turrini C., Tunstall T., Thwaites R., Mauri T., Ragazzi R., Ruggeri P., Hansel T.T., Caramori G. (2019). Biomarkers for Acute Respiratory Distress syndrome and prospects for personalised medicine. J. Inflamm..

[13] Matthay M.A., Ware L.B., Zimmerman G.A. (2012). The acute respiratory distress syndrome. J. Clin. Invest..

[14] Matthay M.A., Zemans R.L., Zimmerman G.A., Arabi Y.M., Beitler J.R., Mercat A., Herridge M., Randolph A.G., Calfee C.S. (2019). Acute respiratory distress syndrome. Nat. Rev. Dis. Primers.

[15] Cardinal-Fernández P., Bajwa E.K., Dominguez-Calvo A., Menéndez J.M., Papazian L., Thompson B.T. (2016). The Presence of Diffuse Alveolar Damage on Open Lung Biopsy Is Associated with Mortality in Patients with Acute Respiratory Distress Syndrome: A Systematic Review and Meta-Analysis. Chest.

[16] Hogan B.L., Barkauskas C.E., Chapman H.A., Epstein J.A., Jain R., Hsia C.C., Niklason L., Calle E., Le A., Randell S.H. (2014). Repair and regeneration of the respiratory system: Complexity, plasticity, and mechanisms of lung stem cell function. Cell. Stem Cell.

[17] Calfee C.S., Janz D.R., Bernard G.R., May A.K., Kangelaris K.N., Matthay M.A., Ware L.B. (2015). Distinct molecular phenotypes of direct vs indirect ARDS in single-center and multicenter studies. Chest.

[18] Calfee C.S., Delucchi K., Parsons P.E., Thompson B.T., Ware L.B., Matthay M.A., NHLBI ARDS Network (2014). Subphenotypes in acute respiratory distress syndrome: Latent class analysis of data from two randomised controlled trials. Lancet Respir. Med..

[19] Famous K.R., Delucchi K., Ware L.B., Kangelaris K.N., Liu K.D., Thompson B.T., Calfee C.S., ARDS Network (2017). Acute Respiratory Distress Syndrome Subphenotypes Respond Differently to Randomized Fluid Management Strategy. Am. J. Respir. Crit. Care Med..

[20] Calfee C.S., Delucchi K.L., Sinha P., Matthay M.A., Hackett J., Shankar-Hari M., McDowell C., Laffey J.G., O’Kane C.M., McAuley D.F. (2018). Acute respiratory distress syndrome subphenotypes and differential response to simvastatin: Secondary analysis of a randomised controlled trial. Lancet Respir. Med..

[21] Delgado-Roche L., Mesta F. (2020). Oxidative stress as key player in severe acute respiratory syndrome coronavirus (SARS-CoV) infection. Arch. Med. Res..

[22] Khomich O.A., Kochetkov S.N., Bartosch B., Ivanov A.V. (2018). Redox biology of respiratory viral infections. Viruses.

[23] Fraternale A., Zara C., De Angelis M., Nencioni L., Palamara A.T., Retini M., Di Mambro T., Magnani M., Crinelli R. (2021). Intracellular Redox-Modulated Pathways as Targets for Effective Approaches in the Treatment of Viral Infection. Int. J. Mol. Sci..

[24] Wu Z., McGoogan J.M. (2020). Characteristics of and Important Lessons from the Coronavirus Disease 2019 (COVID-19) Outbreak in China: Summary of a Report of 72 314 Cases from the Chinese Center for Disease Control and Prevention. J. Am. Med. Assoc..

[25] du Preez H.N., Aldous C., Hayden M.R., Kruger H.G., Lin J. (2022). Pathogenesis of COVID-19 described through the lens of an undersulfated and degraded epithelial and endothelial glycocalyx. FASEB J..

[26] Alam M.S., Czajkowsky D.M. (2022). SARS-CoV-2 infection and oxidative stress: Pathophysiological insight into thrombosis and therapeutic opportunities. Cytokine Growth Factor. Rev..

[27] Li M.Y., Li L., Zhang Y., Wang X.S. (2020). Expression of the SARS-CoV-2 cell receptor gene ACE2 in a wide variety of human tissues. Infect. Dis. Poverty.

[28] Prompetchara E., Ketloy C., Palaga T. (2020). Immune responses in COVID-19 and potential vaccines: Lessons learned from SARS and MERS epidemic. Asian Pac. J. Allergy Immunol..

[29] Karki R., Kanneganti T.D. (2022). Innate immunity, cytokine storm, and inflammatory cell death in COVID-19. J. Transl. Med..

[30] Itri R., Junqueira H.C., Mertins O., Baptista M.S. (2014). Membrane changes under oxidative stress: The impact of oxidized lipids. Biophys. Rev..

[31] Freeman T.L., Swartz T.H. (2020). Targeting the NLRP3 Inflammasome in Severe COVID-19. Front. Immunol..

[32] Zhao N., Di B., Xu L.L. (2021). The NLRP3 inflammasome and COVID-19: Activation, pathogenesis and therapeutic strategies. Cytokine Growth Factor. Rev..

[33] Varga Z., Flammer A.J., Steiger P., Haberecker M., Andermatt R., Zinkernagel A.S., Mehra M.R., Schuepbach R.A., Ruschitzka F., Moch H. (2020). Endothelial cell infection and endotheliitis in COVID-19. Lancet.

[34] Verdecchia P., Cavallini C., Spanevello A., Angeli F. (2020). The pivotal link between ACE2 deficiency and SARS-CoV-2 infection. Eur. J. Intern. Med..

[35] Lei Y., Zhang J., Schiavon C.R., He M., Chen L., Shen H., Zhang Y., Yin Q., Cho Y., Andrade L. (2021). SARS-CoV-2 Spike Protein Impairs Endothelial Function via Downregulation of ACE 2. Circ. Res..

[36] Norooznezhad A.H., Mansouri K. (2021). Endothelial cell dysfunction, coagulation, and angiogenesis in coronavirus disease 2019 (COVID-19). Microvasc. Res.

[37] Zheng Y.Y., Ma Y.T., Zhang J.Y., Xie X. (2020). COVID-19 and the cardiovascular system. Nat. Rev. Cardiol..

[38] Asadi-Pooya A.A., Simani L. (2020). Central nervous system manifestations of COVID-19: A systematic review. J. Neurol. Sci..

[39] Akanchise T., Angelova A. (2023). Potential of Nano-Antioxidants and Nanomedicine for Recovery from Neurological Disorders Linked to Long COVID Syndrome. Antioxidants.

[40] Brower R.G., Matthay M.A., Morris A., Schoenfeld D., Thompson B.T., Wheeler A. (2000). Ventilation with lower tidal volumes as compared with traditional tidal volumes for acute lung injury and the acute respiratory distress syndrome. N. Engl. J. Med..

[41] Fan E., Del Sorbo L., Goligher E.C., Hodgson C.L., Munshi L., Walkey A.J., Adhikari N.K.J., Amato M.B.P., Branson R., Brower R.G. (2017). Official American Thoracic Society/European Society of Intensive Care Medicine/Society of Critical Care Medicine Clinical Practice Guideline: Mechanical Ventilation in Adult Patients with Acute Respiratory Distress Syndrome. Am. J. Respir. Crit. Care Med..

[42] Pelosi P., D’Andrea L., Vitale G., Pesenti A., Gattinoni L. (1994). Vertical gradient of regional lung inflation in adult respiratory distress syndrome. Am. J. Respir. Crit. Care Med..

[43] Guérin C., Reignier J., Richard J.C., Beuret P., Gacouin A., Boulain T., Mercier E., Badet M., Mercat A., Baudin O. (2013). Prone positioning in severe acute respiratory distress syndrome. N. Engl. J. Med..

[44] Manthous C.A., Hall J.B., Olson D., Singh M., Chatila W., Pohlman A., Kushner R., Schmidt G.A., Wood L.D. (1995). Effect of cooling on oxygen consumption in febrile critically ill patients. Am. J. Respir. Crit. Care Med..

[45] Kress J.P., O’Connor M.F., Pohlman A.S., Olson D., Lavoie A., Toledano A., Hall J.B. (1996). Sedation of critically ill patients during mechanical ventilation. A comparison of propofol and midazolam. Am. J. Respir. Crit. Care Med..

[46] Marik P.E., Kaufman D. (1996). The effects of neuromuscular paralysis on systemic and splanchnic oxygen utilization in mechanically ventilated patients. Chest.

[47] Wiedemann H.P., Wheeler A.P., Bernard G.R., Thompson B.T., Hayden D., de Boisblanc B., Connors A.F., Hite R.D., Harabin A.L., National Heart, Lung, and Blood Institute Acute Respiratory Distress Syndrome (ARDS) Clinical Trials Network (2006). Comparison of two fluid-management strategies in acute lung injury. N. Engl. J. Med..

[48] Lewis S.R., Pritchard M.W., Thomas C.M., Smith A.F. (2019). Pharmacological agents for adults with acute respiratory distress syndrome. Cochrane Database Syst. Rev..

[49] Hussain M., Khurram Syed S., Fatima M., Shaukat S., Saadullah M., Alqahtani A.M., Alqahtani T., Bin Emran T., Alamri A.H., Barkat M.Q. (2021). Acute Respiratory Distress Syndrome and COVID-19: A Literature Review. J. Inflamm. Res..

[50] Villar J., Ferrando C., Tusman G., Berra L., Rodríguez-Suárez P., Suárez-Sipmann F. (2021). Unsuccessful and Successful Clinical Trials in Acute Respiratory Distress Syndrome: Addressing Physiology-Based Gaps. Front. Physiol..

[51] Amrein K., Schnedl C., Holl A., Riedl R., Christopher K.B., Pachler C., Urbanic Purkart T., Waltensdorfer A., Münch A., Warnkross H. (2014). Effect of high-dose vitamin D3 on hospital length of stay in critically ill patients with vitamin D deficiency: The VITdAL-ICU randomized clinical trial. J. Am. Med. Assoc..

[52] Ginde A.A., Brower R.G., Caterino J.M., Finck L., Banner-Goodspeed V.M., Grissom C.K., Hayden D., Hough C.L., Hyzy R.C., National Heart, Lung, and Blood Institute PETAL Clinical Trials Network (2019). Early High-Dose Vitamin D3 for Critically Ill, Vitamin D-Deficient Patients. N. Engl. J. Med..

[53] Fowler A.A., Truwit J.D., Hite R.D., Morris P.E., DeWilde C., Priday A., Fisher B., Thacker L.R., Natarajan R., Brophy D.F. (2019). Effect of Vitamin C Infusion on Organ Failure and Biomarkers of Inflammation and Vascular Injury in Patients With Sepsis and Severe Acute Respiratory Failure: The CITRIS-ALI Randomized Clinical Trial. J. Am. Med. Assoc..

[54] Labbani-Motlagh Z., Amini S., Aliannejad R., Sadeghi A., Shafiee G., Heshmat R., Jafary M., Talaschian M., Akhtari M., Jamshidi A. (2022). High-dose Intravenous Vitamin C in Early Stages of Severe Acute Respiratory Syndrome Coronavirus 2 Infection: A Double-blind, Randomized, Controlled Clinical Trial. J. Res. Pharm. Pract..

[55] Mokra D., Mokry J., Barosova R., Hanusrichterova J. (2023). Advances in the Use of N-Acetylcysteine in Chronic Respiratory Diseases. Antioxidants.

[56] Schwalfenberg G.K. (2021). N-Acetylcysteine: A Review of Clinical Usefulness (an Old Drug with New Tricks). J. Nutr. Metab..

[57] Tenório M.C.D.S., Graciliano N.G., Moura F.A., Oliveira A.C.M., Goulart M.O.F. (2021). N-Acetylcysteine (NAC): Impacts on Human Health. Antioxidants.

[58] Zhitkovich A. (2019). N-Acetylcysteine: Antioxidant, Aldehyde Scavenger, and More. Chem. Res. Toxicol..

[59] Izquierdo-Alonso J.L., Pérez-Rial S., Rivera C.G., Peces-Barba G. (2022). N-acetylcysteine for prevention and treatment of COVID-19: Current state of evidence and future directions. J. Infect. Public. Health.

[60] Ji L., Liu R., Zhang X.D., Chen H.L., Bai H., Wang X., Zhao H.L., Liang X., Hai C.X. (2010). N-acetylcysteine attenuates phosgene-induced acute lung injury via up-regulation of Nrf2 expression. Inhal. Toxicol..

[61] Oter S., Jin S., Cucullo L., Dorman H.J. (2012). Oxidants and antioxidants: Friends or foes?. Oxid. Antioxid. Med. Sci..

[62] Milara J., Martínez-Expósito F., Montero P., Roger I., Bayarri M.A., Ribera P., Oishi-Konari M.N., Alba-García J.R., Zapater E., Cortijo J. (2022). N-acetylcysteine Reduces Inflammasome Activation Induced by SARS-CoV-2 Proteins In Vitro. Int. J. Mol. Sci..

[63] Hashimoto S., Gon Y., Matsumoto K., Takeshita I., Horie T. (2001). N-acetylcysteine attenuates TNF-alpha-induced p38 MAP kinase activation and p38 MAP kinase-mediated IL-8 production by human pulmonary vascular endothelial cells. Br. J. Pharmacol..

[64] Chen J., Reheman A., Gushiken F.C., Nolasco L., Fu X., Moake J.L., Ni H., López J.A. (2011). N-acetylcysteine reduces the size and activity of von Willebrand factor in human plasma and mice. J. Clin. Invest..

[65] Martinez de Lizarrondo S., Gakuba C., Herbig B.A., Repessé Y., Ali C., Denis C.V., Lenting P.J., Touzé E., Diamond S.L., Vivien D. (2017). Potent Thrombolytic Effect of N-Acetylcysteine on Arterial Thrombi. Circulation.

[66] Morris G., Bortolasci C.C., Puri B.K., Olive L., Marx W., O’Neil A., Athan E., Carvalho A., Maes M., Walder K. (2021). Preventing the development of severe COVID-19 by modifying immunothrombosis. Life Sci..

[67] Cazzola M., Rogliani P., Salvi S.S., Ora J., Matera M.G. (2021). Use of Thiols in the Treatment of COVID-19: Current Evidence. Lung.

[68] Araki S., Dobashi K., Kubo K., Yamamoto Y., Asayama K., Shirahata A. (2006). N-acetylcysteine attenuates TNF-α induced changes in secretion of interleukin-6, plasminogen activator inhibitor-1 and adiponectin from 3T3-L1 adipocytes. Life Sci..

[69] Zuo Y., Warnock M., Harbaugh A., Yalavarthi S., Gockman K., Zuo M., Madison J.A., Knight J.S., Kanthi Y., Lawrence D.A. (2021). Plasma tissue plasminogen activator and plasminogen activator inhibitor-1 in hospitalized COVID-19 patients. Sci. Rep..

[70] Ullian M.E., Gelasco A.K., Fitzgibbon W.R., Beck C.N., Morinelli T.A. (2005). N-acetylcysteine decreases angiotensin II receptor binding in vascular smooth muscle cells. J. Am. Soc. Nephrol..

[71] Capettini L.S., Montecucco F., Mach F., Stergiopulos N., Santos R.A., da Silva R.F. (2012). Role of renin-angiotensin system in inflammation, immunity and aging. Curr. Pharm. Des..

[72] McCarty M.F., DiNicolantonio J.J. (2020). Nutraceuticals have potential for boosting the type 1 interferon response to RNA viruses including influenza and coronavirus. Prog. Cardiovasc. Dis..

[73] Hati S., Bhattacharyya S. (2020). Impact of thiol-disulfide balance on the binding of Covid-19 spike protein with Angiotensin Converting Enzyme 2 receptor. ACS. Omega.

[74] Matute-Bello G., Frevert C.W., Martin T.R. (2008). Animal models of acute lung injury. Am. J. Physiol. Lung Cell. Mol. Physiol..

[75] Mokra D., Mikolka P., Kosutova P., Calkovska A. (2020). Experimental models of acute lung injury: Their advantages and limitations. Acta Med. Martin..

[76] Blondonnet R., Constantin J.M., Sapin V., Jabaudon M. (2016). A Pathophysiologic Approach to Biomarkers in Acute Respiratory Distress Syndrome. Dis. Markers.

[77] Xu J.F., Qu J.M., Li H.P. (2011). N-Acetylcysteine modulates acute lung injury induced by Pseudomonas aeruginosa in rats. Clin. Exp. Pharmacol. Physiol..

[78] Leal A.P.F., Nieto Marín V., Cabistany V.V., Morales J., Buccini D.F., Franco O.L. (2024). Applicability of mouse models for induction of severe acute lung injury. Pulm. Pharmacol. Ther..

[79] Demiralay R., Gürsan N., Ozbilim G., Erdogan G., Demirci E. (2006). Comparison of the effects of erdosteine and N-acetylcysteine on apoptosis regulation in endotoxin-induced acute lung injury. J. Appl. Toxicol..

[80] Lima Trajano E.T., Sternberg C., Caetano M., Santos Silva M.A., Porto L.C., Santos J.C., Ribeiro M.L., Magalhães C.B., Zin W.A., Benjamim C.F. (2011). Endotoxin-induced acute lung injury is dependent upon oxidative response. Inhal. Toxicol..

[81] Chen H., Ma N., Song X., Wei G., Zhang H., Liu J., Shen X., Zhuge X., Chang G. (2022). Protective Effects of N-Acetylcysteine on Lipopolysaccharide-Induced Respiratory Inflammation and Oxidative Stress. Antioxidants.

[82] Kolomaznik M., Mikolka P., Hanusrichterova J., Kosutova P., Matasova K., Mokra D., Calkovska A. (2021). N-Acetylcysteine in Mechanically Ventilated Rats with Lipopolysaccharide-Induced Acute Respiratory Distress Syndrome: The Effect of Intravenous Dose on Oxidative Damage and Inflammation. Biomedicines.

[83] Mokra D., Calkovska A. (2017). Experimental models of acute lung injury in the newborns. Physiol. Res..

[84] Chen C., Guan X., Quinn D.A., Ouyang B. (2015). N-Acetylcysteine Inhibits Ventilation-Induced Collagen Accumulation in the Rat Lung. Tohoku J. Exp. Med..

[85] Cao C., Zhang L., Shen J. (2022). Phosgene-Induced acute lung injury: Approaches for mechanism-based treatment strategies. Front. Immunol..

[86] Rendell R., Fairhall S., Graham S., Rutter S., Auton P., Smith A., Perrott R., Jugg B. (2018). Assessment of N-acetylcysteine as a therapy for phosgene-induced acute lung injury. Toxicol. Lett..

[87] Mokra D., Drgova A., Petras M., Mokry J., Antosova M., Calkovska A. (2015). N-acetylcysteine alleviates the meconium-induced acute lung injury. Adv. Exp. Med. Biol..

[88] Mokra D., Drgova A., Mokry J., Antosova M., Durdik P., Calkovska A. (2015). N-acetylcysteine effectively diminished meconium-induced oxidative stress in adult rabbits. J. Physiol. Pharmacol..

[89] Mokra D., Tonhajzerova I., Pistekova H., Visnovcova Z., Drgova A., Mokry J., Calkovska A. (2015). Cardiovascular effects of N-acetylcysteine in meconium-induced acute lung injury. Adv. Exp. Med. Biol..

[90] Požgain Z., Kristek D., Lovrić I., Kondža G., Jelavić M., Kocur J., Danilović M. (2018). Pulmonary contusions after blunt chest trauma: Clinical significance and evaluation of patient management. Eur. J. Trauma. Emerg. Surg..

[91] Türüt H., Ciralik H., Kilinc M., Ozbag D., Imrek S.S. (2009). Effects of early administration of dexamethasone, N-acetylcysteine and aprotinin on inflammatory and oxidant-antioxidant status after lung contusion in rats. Injury.

[92] Chavko M., Adeeb S., Ahlers S.T., McCarron R.M. (2009). Attenuation of pulmonary inflammation after exposure to blast overpressure by N-acetylcysteine amide. Shock.

[93] Inci I., Zhai W., Arni S., Hillinger S., Vogt P., Weder W. (2007). N-acetylcysteine attenuates lung ischemia-reperfusion injury after lung transplantation. Ann. Thorac. Surg..

[94] Forgiarini L.F., Forgiarini L.A., da Rosa D.P., Silva M.B., Mariano R., Paludo Ade O., Andrade C.F. (2014). N-acetylcysteine administration confers lung protection in different phases of lung ischaemia-reperfusion injury. Interact. Cardiovasc. Thorac. Surg..

[95] Kao S.J., Wang D., Lin H.I., Chen H.I. (2006). N-acetylcysteine abrogates acute lung injury induced by endotoxin. Clin. Exp. Pharmacol. Physiol..

[96] Baboolal H.A., Ichinose F., Ullrich R., Kawai N., Bloch K.D., Zapol W.M. (2002). Reactive oxygen species scavengers attenuate endotoxin-induced impairment of hypoxic pulmonary vasoconstriction in mice. Anesthesiology.

[97] Demiralay R., Gürsan N., Erdem H. (2006). Regulation of sepsis-induced apoptosis of pulmonary cells by posttreatment of erdosteine and N-aceylcysteine. Toxicology.

[98] Choi J.S., Lee H.S., Seo K.H., Na J.O., Kim Y.H., Uh S.T., Park C.S., Oh M.H., Lee S.H., Kim Y.T. (2012). The effect of post-treatment N-acetylcysteine in LPS-induced acute lung injury of rats. Tuberc. Respir. Dis..

[99] Campos R., Shimizu M.H., Volpini R.A., de Bragança A.C., Andrade L., Lopes F.D., Olivo C., Canale D., Seguro A.C. (2012). N-acetylcysteine prevents pulmonary edema and acute kidney injury in rats with sepsis submitted to mechanical ventilation. Am. J. Physiol. Lung Cell. Mol. Physiol..

[100] Le J.W., Sun M., Zhu J.H., Fan H. (2022). Protective effect of N-acetylcysteine on acute lung injury in septic rats by inhibiting inflammation, oxidation, and apoptosis. Iran. J. Basic. Med. Sci..

[101] Ozdulger A., Cinel I., Koksel O., Cinel L., Avlan D., Unlu A., Okcu H., Dikmengil M., Oral U. (2003). The protective effect of N-acetylcysteine on apoptotic lung injury in cecal ligation and puncture-induced sepsis model. Shock.

[102] Gürer A., Ozdoğan M., Gökakin A.K., Gömceli I., Gülbahar O., Arikök A.T., Kulaçoğlu H., Aydin R. (2009). Tissue oxidative stress level and remote organ injury in two-hit trauma model of sequential burn injury and peritoneal sepsis are attenuated with N-acetylcysteine treatment in rats. Ulus. Travma Acil Cerrahi Derg..

[103] Pfeifer R., Andruszkow J.H., Busch D., Hoepken M., Barkatali B.M., Horst K., Pape H.C., Hildebrand F. (2015). Development of a standardized trauma-related lung injury model. J. Surg. Res..

[104] Alkan A., Eroğlu F., Eroğlu E., Ergin C., Cerçi C., Alsancak G. (2006). Protective effects of N-acetylcysteine and erdosteine on hemorrhagic shock-induced acute lung injury. Eur. J. Emerg. Med..

[105] Lee J.H., Jo Y.H., Kim K., Lee J.H., Rim K.P., Kwon W.Y., Suh G.J., Rhee J.E. (2013). Effect of N-acetylcysteine (NAC) on acute lung injury and acute kidney injury in hemorrhagic shock. Resuscitation.

[106] Saad K.R., Saad P.F., Dantas Filho L., Brito J.M., Koike M.K., Zanoni F.L., Dolhnikoff M., Montero E.F. (2013). Pulmonary impact of N-acetylcysteine in a controlled hemorrhagic shock model in rats. J. Surg. Res..

[107] Azarkish F., Nematbakhsh M., Fazilati M., Talebi A., Pilehvarian A.A., Pezeshki Z., Moeini M., Mansouri A., Safari T. (2013). N-acetylcysteine Prevents Kidney and Lung Disturbances in Renal Ischemia/Reperfusion Injury in Rat. Int. J. Prev. Med..

[108] Koksel O., Cinel I., Tamer L., Cinel L., Ozdulger A., Kanik A., Ercan B., Oral U. (2004). N-acetylcysteine inhibits peroxynitrite-mediated damage in oleic acid-induced lung injury. Pulm. Pharmacol. Ther..

[109] Kumar S., Bhagat P., Pandey S., Pandey R. (2022). The Role of Antioxidant Agent (N-Acetylcysteine) in Oleic Acid-Induced Acute Lung Injury in a Rat Model. Cureus.

[110] Bhatia M., Wong F.L., Cao Y., Lau H.Y., Huang J., Puneet P., Chevali L. (2005). Pathophysiology of acute pancreatitis. Pancreatology.

[111] Yubero S., Ramudo L., Manso M.A., Collía F., De Dios I. (2012). Evaluation of N-acetylcysteine treatment in acute pancreatitis-induced lung injury. Inflamm. Res..

[112] Suter P.M., Domenighetti G., Schaller M.D., Laverrière M.C., Ritz R., Perret C. (1994). N-acetylcysteine enhances recovery from acute lung injury in man. A randomized, double-blind, placebo-controlled clinical study. Chest.

[113] Bernard G.R., Wheeler A.P., Arons M.M., Morris P.E., Paz H.L., Russell J.A., Wright P.E. (1997). A trial of antioxidants N-acetylcysteine and procysteine in ARDS. The Antioxidant in ARDS Study Group. Chest.

[114] Domenighetti G., Suter P.M., Schaller M.D., Ritz R., Perret C. (1997). Treatment with N-acetylcysteine during acute respiratory distress syndrome: A randomized, double-blind, placebo-controlled clinical study. J. Crit. Care.

[115] Soltan-Sharifi M.S., Mojtahedzadeh M., Najafi A., Reza Khajavi M., Reza Rouini M., Moradi M., Mohammadirad A., Abdollahi M. (2007). Improvement by N-acetylcysteine of acute respiratory distress syndrome through increasing intracellular glutathione, and extracellular thiol molecules and anti-oxidant power: Evidence for underlying toxicological mechanisms. Hum. Exp. Toxicol..

[116] Zhang Q., Ju Y., Ma Y., Wang T. (2018). N-acetylcysteine improves oxidative stress and inflammatory response in patients with community acquired pneumonia: A randomized controlled trial. Medicine.

[117] Sharafkhah M., Abdolrazaghnejad A., Zarinfar N., Mohammadbeigi A., Massoudifar A., Abaszadeh S. (2018). Safety and efficacy of N-acetyl-cysteine for prophylaxis of ventilator-associated pneumonia: A randomized, double blind, placebo-controlled clinical trial. Med. Gas. Res..

[118] Ghorbi M., Rashidi M., Olapour A., Javaherforooshzadeh F., Akhondzadeh R. (2021). Effect of N-Acetylcysteine on the treatment of acute respiratory distress syndrome in mechanically ventilated patients admitted to the intensive care unit. Med. J. Islam. Repub. Iran..

[119] Mohanty R.R., Padhy B.M., Das S., Meher B.R. (2021). Therapeutic potential of N-acetyl cysteine (NAC) in preventing cytokine storm in COVID-19: Review of current evidence. Eur. Rev. Med. Pharmacol. Sci..

[120] Wong K.K., Lee S.W.H., Kua K.P. (2021). N-Acetylcysteine as Adjuvant Therapy for COVID-19—A Perspective on the Current State of the Evidence. J. Inflamm. Res..

[121] Di Marco F., Foti G., Corsico A.G. (2022). Where are we with the use of N-acetylcysteine as a preventive and adjuvant treatment for COVID-19?. Eur. Rev. Med. Pharmacol. Sci..

[122] Liu T.H., Wu J.Y., Huang P.Y., Tsai Y.W., Hsu W.H., Chuang M.H., Tang H.J., Lai C.C. (2024). Clinical efficacy of N-acetylcysteine for COVID-19: A systematic review and meta-analysis of randomized controlled trials. Heliyon.

[123] Ibrahim H., Perl A., Smith D., Lewis T., Kon Z., Goldenberg R., Yarta K., Staniloae C., Williams M. (2020). Therapeutic blockade of inflammation in severe COVID-19 infection with intravenous N-acetylcysteine. Clin. Immunol..

[124] de Alencar J.C.G., Moreira C.L., Müller A.D., Chaves C.E., Fukuhara M.A., da Silva E.A., Miyamoto M.F.S., Pinto V.B., Bueno C.G., Lazar Neto F. (2021). Double-blind, Randomized, Placebo-controlled Trial with N-acetylcysteine for Treatment of Severe Acute Respiratory Syndrome Caused by Coronavirus Disease 2019 (COVID-19). Clin. Infect. Dis..

[125] Taher A., Lashgari M., Sedighi L., Rahimi-Bashar F., Poorolajal J., Mehrpooya M. (2021). A pilot study on intravenous N-Acetylcysteine treatment in patients with mild-to-moderate COVID19-associated acute respiratory distress syndrome. Pharmacol. Rep..

[126] Faverio P., Rebora P., Rossi E., Del Giudice S., Montanelli F., Garzillo L., Busnelli S., Luppi F., Valsecchi M.G., Pesci A. (2021). Impact of N-acetyl-l-cysteine on SARS-CoV-2 pneumonia and its sequelae: Results from a large cohort study. ERJ. Open Res..

[127] Avdeev S.N., Gaynitdinova V.V., Merzhoeva Z.M., Berikkhanov Z.G. (2022). N-acetylcysteine for the treatment of COVID-19 among hospitalized patients. J. Infect..

[128] Assimakopoulos S.F., Aretha D., Komninos D., Dimitropoulou D., Lagadinou M., Leonidou L., Oikonomou I., Mouzaki A., Marangos M. (2021). N-acetyl-cysteine reduces the risk for mechanical ventilation and mortality in patients with COVID-19 pneumonia: A two-center retrospective cohort study. Infect. Dis..

[129] Izquierdo J.L., Soriano J.B., González Y., Lumbreras S., Ancochea J., Echeverry C., Rodríguez J.M. (2022). Use of N-Acetylcysteine at high doses as an oral treatment for patients hospitalized with COVID-19. Sci. Prog..

[130] Chavarría A.P., Vázquez R.R.V., Cherit J.G.D., Bello H.H., Suastegui H.C., Moreno-Castañeda L., Alanís Estrada G., Hernández F., González-Marcos O., Saucedo-Orozco H. (2021). Antioxidants and pentoxifylline as coadjuvant measures to standard therapy to improve prognosis of patients with pneumonia by COVID-19. Comput. Struct. Biotechnol. J..

[131] Atefi N., Goodarzi A., Riahi T., Khodabandehloo N., Talebi Taher M., Najar Nobari N., Seirafianpour F., Mahdi Z., Baghestani A., Valizadeh R. (2023). Evaluation of the efficacy and safety of oral N-acetylcysteine in patients with COVID-19 receiving the routine antiviral and hydroxychloroquine protocol: A randomized controlled clinical trial. Immun. Inflamm. Dis..

[132] Panahi Y., Ghanei M., Rahimi M., Samim A., Vahedian-Azimi A., Atkin S.L., Sahebkar A. (2023). Evaluation the efficacy and safety of N-acetylcysteine inhalation spray in controlling the symptoms of patients with COVID-19: An open-label randomized controlled clinical trial. J. Med. Virol..

[133] Shi Z., Puyo C.A. (2020). N-acetylcysteine to combat COVID-19: An evidence review. Ther. Clin. Risk Manag..

[134] Lai K.Y., Au S.Y., Sin K.C., Yung S.K., Leung A.K.H. (2024). High-dose N-acetylcysteine in an immunocompromised patient with COVID-19: A case report. Hong. Kong Med. J..

[135] Liu Y., Wang M., Luo G., Qian X., Wu C., Zhang Y., Chen B., Leung E.L., Tang Y. (2020). Experience of N-acetylcysteine airway management in the successful treatment of one case of critical condition with COVID-19: A case report. Medicine.

[136] Bellone S., Siegel E.R., Santin A.D. (2025). N-acetylcysteine (NAC) supplementation improves dyspnea and may normalize von Willebrand plasma levels in gynecologic patients with Post-Acute-COVID-Sequela (PASC)/Long COVID. Gynecol. Oncol. Rep..

[137] Carothers C., Birrer K., Vo M. (2020). Acetylcysteine for the Treatment of Suspected Remdesivir-Associated Acute Liver Failure in COVID-19: A Case Series. Pharmacotherapy.

[138] Lu X., Ma Y., He J., Li Y., Zhu H., Yu X. (2019). N-acetylcysteine for adults with acute respiratory distress syndrome: A meta-analysis of randomized controlled trials. Hong. Kong J. Emerg. Med..

[139] Qiao Q., Liu X., Yang T., Cui K., Kong L., Yang C., Zhang Z. (2021). Nanomedicine for acute respiratory distress syndrome: The latest application, targeting strategy, and rational design. Acta Pharm. Sin. B.

[140] Moss D.M., Curley P., Kinvig H., Hoskins C., Owen A. (2018). The Biological Challenges and Pharmacological Opportunities of Orally Administered Nanomedicine Delivery. Expert. Rev. Gastroenterol. Hepatol..

[141] Liu Y., Zhou S., Xiang D., Ju L., Shen D., Wang X., Wang Y. (2021). Friend or Foe? The Roles of Antioxidants in Acute Lung Injury. Antioxidants.

[142] Calabrese E.J., Baldwin L.A. (2003). Inorganics and hormesis. Crit. Rev. Toxicol..

[143] Holdiness M.R. (1991). Clinical pharmacokinetics of N-acetylcysteine. Clin. Pharmacokinet..

[144] Lana J.F.S.D., Lana A.V.S.D., Rodrigues Q.S., Santos G.S., Navani R., Navani A., da Fonseca L.F., Azzini G.O.M., Setti T., Mosaner T. (2021). Nebulization of glutathione and N-Acetylcysteine as an adjuvant therapy for COVID-19 onset. Adv. Redox Res..

[145] Delić N., Matetic A., Domjanović J., Kljaković-Gašpić T., Šarić L., Ilić D., Došenović S., Domazet J., Kovač R., Runjić F. (2022). Effects of Different Inhalation Therapy on Ventilator-Associated Pneumonia in Ventilated COVID-19 Patients: A Randomized Controlled Trial. Microorganisms.

[146] Murgia X., de Souza Carvalho C., Lehr C.M. (2014). Overcoming the pulmonary barrier: New insights to improve the efficiency of inhaled therapeutics. Eur. J. Nanomed..

[147] Ritter C., Andrades M.E., Reinke A., Menna-Barreto S., Moreira J.C., Dal-Pizzol F. (2004). Treatment with N-acetylcysteine plus deferoxamine protects rats against oxidative stress and improves survival in sepsis. Crit. Care Med..

[148] Ritter C., da Cunha A.A., Echer I.C., Andrades M., Reinke A., Lucchiari N., Rocha J., Streck E.L., Menna-Barreto S., Moreira J.C. (2006). Effects of N-acetylcysteine plus deferoxamine in lipopolysaccharide-induced acute lung injury in the rat. Crit. Care Med..

[149] Kolomaznik M., Hanusrichterova J., Mikolka P., Kosutova P., Vatecha M., Zila I., Mokra D., Calkovska A. (2023). Efficiency of exogenous surfactant combined with intravenous N-acetylcysteine in two-hit rodent model of ARDS. Respir. Physiol. Neurobiol..

[150] Kopincová J., Mokrá D., Mikolka P., Kolomazník M., Čalkovská A. (2014). N-acetylcysteine advancement of surfactant therapy in experimental meconium aspiration syndrome: Possible mechanisms. Physiol. Res..

[151] Mikolka P., Kopincova J., Mikusiakova L.T., Kosutova P., Calkovska A., Mokra D. (2016). Antiinflammatory Effect of N-Acetylcysteine Combined with Exogenous Surfactant in Meconium-Induced Lung Injury. Adv. Exp. Med. Biol..

[152] Kopincova J., Kolomaznik M., Mikolka P., Kosutova P., Topercerova J., Matasova K., Calkovska A., Mokra D. (2019). Recombinant Human Superoxide Dismutase and N-Acetylcysteine Addition to Exogenous Surfactant in the Treatment of Meconium Aspiration Syndrome. Molecules.

[153] Wigenstam E., Koch B., Bucht A., Jonasson S. (2015). N-acetyl cysteine improves the effects of corticosteroids in a mouse model of chlorine-induced acute lung injury. Toxicology.

[154] Martini N., Singla P., Arbuckle E., Goyal G., Liu Q., Santos-Zabala M.L., Zainah H. (2023). SARS-CoV-2-Induced Autoimmune Hepatitis. Cureus.

[155] Leme A.S., Lichtenstein A., Arantes-Costa F.M., Landucci E.C., Martins M.A. (2002). Acute lung injury in experimental pancreatitis in rats: Pulmonary protective effects of crotapotin and N-acetylcysteine. Shock.

[156] Guo D.W., Wang C.Y., Shih H.C. (2019). N-acetylcysteine and atorvastatin alleviates lung injury due to ischemia-reperfusion injury in rats. J. Chin. Med. Assoc..

[157] Song Q., Lin L., Chen L., Cheng L., Zhong W. (2020). Co-administration of N-acetylcysteine and dexmedetomidine plays a synergistic effect on protection of LPS-induced acute lung injury via correcting Th1/Th2/Th17 cytokines imbalance. Clin. Exp. Pharmacol. Physiol..

[158] Warren B., Royall N., Smith H., Bhullar I.S. (2016). Novel Treatment of Acute Respiratory Distress Syndrome after Chlorine Gas Inhalation Injury. Am. Surg..

[159] Dube K.M., Ditch K.L., Hills L. (2017). Use of Nebulized Heparin, Nebulized N-Acetylcysteine, and Nebulized Epoprostenol in a Patient with Smoke Inhalational Injury and Acute Respiratory Distress Syndrome. J. Pharm. Pract..

[160] Shaikh N., Chanda A.H., Rahman M.A., Nainthramveetil M.M., Kumar A., Mathias R.M., Nashwan A.J. (2022). Inhalational injury and use of heparin & N-acetylcysteine nebulization: A case report. Respir. Med. Case Rep..

[161] Kim S., Kim S.Y., Rho S.J., Kim S.H., Song S.H., Kim C.H., Lee H., Kim S.K. (2021). Biocompatible N-acetyl-nanoconstruct alleviates lipopolysaccharide-induced acute lung injury in vivo. Sci. Rep..

[162] Mitsopoulos P., Omri A., Alipour M., Vermeulen N., Smith M.G., Suntres Z.E. (2008). Effectiveness of liposomal-N-acetylcysteine against LPS-induced lung injuries in rodents. Int. J. Pharm..

[163] Mitsopoulos P., Suntres Z.E. (2011). Protective Effects of Liposomal N-Acetylcysteine against Paraquat-Induced Cytotoxicity and Gene Expression. J. Toxicol..

[164] Wang J., Yang H., Zheng D., Sun Y., An L., Li G., Zhao Z. (2023). Integrating network pharmacology and pharmacological evaluation to reveal the therapeutic effects and potential mechanism of S-allylmercapto-N-acetylcysteine on acute respiratory distress syndrome. Int. Immunopharmacol..

